# Both ADP-Ribosyl-Binding and Hydrolase Activities of the Alphavirus nsP3 Macrodomain Affect Neurovirulence in Mice

**DOI:** 10.1128/mBio.03253-19

**Published:** 2020-02-11

**Authors:** Rachy Abraham, Robert L. McPherson, Morgan Dasovich, Mohsen Badiee, Anthony K. L. Leung, Diane E. Griffin

**Affiliations:** a W. Harry Feinstone Department of Molecular Microbiology and Immunology, Bloomberg School of Public Health, Johns Hopkins University, Baltimore, Maryland, USA; b Department of Biochemistry and Molecular Biology, Bloomberg School of Public Health, Johns Hopkins University, Baltimore, Maryland, USA; c Department of Chemistry, Krieger School of Arts and Sciences, Johns Hopkins University, Baltimore, Maryland, USA; d Department of Molecular Biology and Genetics, School of Medicine, Johns Hopkins University, Baltimore, Maryland, USA; e Department of Oncology, School of Medicine, Johns Hopkins University, Baltimore, Maryland, USA; Columbia University College of Physicians and Surgeons

**Keywords:** alphavirus, macrodomain, ADP-ribosyl hydrolase, ADP-ribosyl-binding activity, innate immune response, antibody, encephalomyelitis, Sindbis virus, nsP3

## Abstract

Macrodomain (MD), a highly conserved protein fold present in a subset of plus-strand RNA viruses, binds to and hydrolyzes ADP-ribose (ADPr) from ADP-ribosylated proteins. ADPr-binding by the alphavirus nonstructural protein 3 (nsP3) MD is necessary for the initiation of virus replication in neural cells, whereas hydrolase activity facilitates replication complex amplification. To determine the importance of these activities for pathogenesis of alphavirus encephalomyelitis, mutations were introduced into the nsP3 MD of Sindbis virus (SINV), and the effects on ADPr binding and hydrolase activities, virus replication, immune responses, and disease were assessed. Elimination of ADPr-binding and hydrolase activities (G32E) severely impaired *in vitro* replication of SINV in neural cells and *in vivo* replication in the central nervous systems of 2-week-old mice with reversion to wild type (WT) (G) or selection of a less compromising change (S) during replication. SINVs with decreased binding and hydrolase activities (G32S and G32A) or with hydrolase deficiency combined with better ADPr-binding (Y114A) were less virulent than WT virus. Compared to the WT, the G32S virus replicated less well in both the brain and spinal cord, induced similar innate responses, and caused less severe disease with full recovery of survivors, whereas the Y114A virus replicated well, induced higher expression of interferon-stimulated and NF-κB-induced genes, and was cleared more slowly from the spinal cord with persistent paralysis in survivors. Therefore, MD function was important for neural cell replication both *in vitro* and *in vivo* and determined the outcome from alphavirus encephalomyelitis in mice.

## Abstract (precis)

Viral encephalomyelitis is an important cause of long-term disability, as well as acute fatal disease. Identifying viral determinants of outcome helps in assessing disease severity and developing new treatments. Mosquito-borne alphaviruses infect neurons and cause fatal disease in mice. The highly conserved macrodomain of nonstructural protein 3 binds and can remove ADP-ribose (ADPr) from ADP-ribosylated proteins. To determine the importance of these functions for virulence, recombinant mutant viruses were produced. If macrodomain mutations eliminated ADPr-binding or hydrolase activity, viruses did not grow. If the binding and hydrolase activities were impaired, the viruses grew less well than the wild-type virus, induced similar innate responses, and caused less severe disease, and most of the infected mice recovered. If binding was improved, but hydrolase activity was decreased, the virus replicated well and induced greater innate responses than did the WT, but clearance from the nervous system was impaired, and mice remained paralyzed. Therefore, macrodomain function determined the outcome of alphavirus encephalomyelitis.

## INTRODUCTION

Alphaviruses are icosahedral, enveloped, message-sense RNA viruses that are transmitted by mosquitoes and cause fever, rash, myalgia, arthralgia, and encephalomyelitis (1–4). Determinants of alphavirus virulence are present in both the structural and nonstructural proteins with changes in surface glycoprotein E2 and nonstructural protein 3 (nsP3) most often associated with altered neurovirulence in mice (5–10). E2 changes affect binding and entry into target cells, while the function(s) of nsP3 important for virulence has been more difficult to discern (11, 12).

Alphavirus nsP3 has three domains: a phosphorylated C-terminal unstructured hypervariable domain, a zinc-binding alphavirus unique domain and an N-terminal macrodomain (MD). The hypervariable domain interacts with multiple cellular proteins important for virus replication (13–18), and the zinc-binding domain has a role in the synthesis of viral RNA (19–22). The highly conserved MD consists of a central beta sheet surrounded by four to six helices, a protein fold that exists in all kingdoms of life, including a few families of plus-strand RNA viruses (23–27). MDs bind ADP-ribose (ADPr) on proteins posttranslationally modified by either monomers or polymers of ADPr, a modification known as ADP-ribosylation. ADP-ribosylation is catalyzed by enzymes of the ADP-ribosyltransferase diphtheria toxin–like family of proteins commonly known as poly(ADP-ribose) polymerases (PARPs) that transfer ADPr from NAD^+^ to specific residues on target proteins (28–34).

MDs are also present in the nonstructural proteins of coronaviruses, hepatitis E virus, and rubella virus (25, 26, 35), with roles in determining RNA synthesis, replication, and virulence (36–40). Viral MDs are of the Macro D subclass that also has ADP-ribosylhydrolase activity and therefore can remove ADP-ribosylation from modified proteins (26, 41–45). Because several PARPs are regulated by interferon (IFN), have antiviral properties, and are under evolutionary diversifying selection (46–49), it has been postulated that the primary function of viral MDs is to counter the host response to infection by removing ADPr from modified host proteins (50, 51). However, chikungunya virus (CHIKV) with nsP3 MD mutations resulting in little or no hydrolase or binding activity is not viable in either mammalian or insect cells, indicating an important role for the alphavirus MD in virus replication (43). CHIKV with mutations that reduced, but did not eliminate, ADPr-binding or hydrolase activity were viable, but replication in neural cells and virulence for newborn mice were impaired. Further *in vitro* studies showed that ADPr-binding is necessary for initiation of replication in neural cells, whereas hydrolase activity facilitates the amplification of replication complexes (37). However, the effects on neurovirulence have received limited attention.

To analyze the importance of nsP3 MD function for neurovirulence and the induction of innate and adaptive antiviral immune responses in the CNS, we have introduced similar mutations into the nsP3 MD of the TE strain of SINV, a well-characterized mouse model of alphavirus encephalomyelitis that causes fatal disease in 2-week-old mice (5, 52–54). Previous studies have shown that mutation D10A in the ADPr-binding site is not tolerated, while mutation N24A results in viable virus with impaired shutoff of host protein synthesis and decreased virulence (55, 56). In the current studies, multiple SINV MD mutants were characterized biochemically and assessed for replication in neural cells, neurovirulence, and immune responses in the central nervous system (CNS) and showed that ADPr-binding and hydrolase functions of the nsP3 MD differentially affect the outcome of CNS infection.

## RESULTS

### Development of mutations in the nsP3 MD and characterization of the effects on ADPr-binding and hydrolase activities.

**FIG 1:**
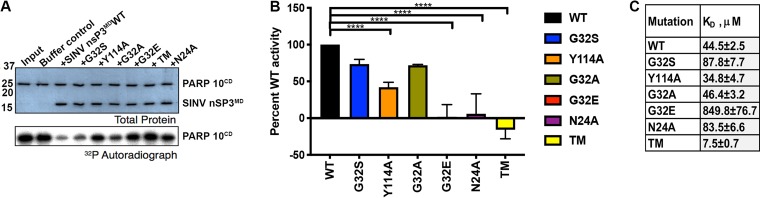
ADP-ribosyl-binding and hydrolase activities of SINV nsP3^MD^ mutants. (A) Representative image of results from the PARP10 catalytic domain (PARP10^CD^) demodification assay. PARP10^CD^ was incubated with ^32^P-NAD^+^ to generate ^32^P-MARylated PARP10^CD^, which was incubated with buffer alone, nsP3 MDs from WT and mutants for 1 h at 37°C, followed by analysis by SDS-PAGE and autoradiography. Changes in the intensity of ^32^P-MARylated PARP10^CD^ in samples containing nsP3^MD^ from WT and mutants were quantified. (B) Quantitative representation of MAR hydrolase activity of nsP3 MD mutants relative to WT. Assays were performed in triplicate, buffer control was subtracted, and values were normalized to the activity levels of nsP3 MD WT. The data are presented as the percent WT activity values obtained from three independent experiments. Significance was determined by one-way ANOVA with Dunnett’s multiple-comparison test. ****, *P* < 0.0001 (WT versus N24A, G32E, TM [G32E/I113R/Y114N], and Y114A). (C) Quantification of ADPr-binding in *K_D_* (μM) from three runs of microscale thermophoresis (MST). Defined length PAR labeled on the 1″ terminus with Cy5 (10 nM) was incubated with 2-fold serial dilutions (diluted down from 0.5 to 1 mM stock concentration to 15 to 30 nM) of SINV WT and mutant MDs. MST was measured using a Monolith NT.115 (NanoTemper) at 80% excitation power and 20% MST power. The data are shown as the mean normalized fluorescence ± the SD.

Based on information gained from the structure of the alphavirus nsP3 MD (27) and previous mutational analyses of the binding and hydrolase functions of the CHIKV MD (43), we introduced alanine substitutions into highly conserved amino acids in the ADPr-binding site (positions 24 and 114) and catalytic hydrolase loop (positions 24 and 32) to alter these MD functions. N24 is within the hydrolase loop and coordinates binding to the distal ribose, as does Y114. G32 is also in the hydrolase loop, and previous studies showed that amino acid substitutions at the equivalent of this position can fine-tune hydrolase activity (27, 41, 43, 44, 50, 57). Purified wild-type (WT) and recombinant nsP3 MD mutant N24A, G32S, G32A, G32E, Y114A, and G32E/I113R/Y114N (triple-mutant [TM]) strains were assessed for MAR hydrolase activity (Fig. 1A and B) and ADPr-binding (Fig. 1C) (43, 58).

To measure hydrolase activity, the catalytic domain of PARP10 (PARP10^CD^) was incubated with ^32^P-NAD^+^ to generate ^32^P-mono-ADP-ribosylated (MARylated) PARP10^CD^ as a substrate for incubation with buffer alone, WT, or mutant nsP3 MDs for 1 h at 37°C, followed by analysis by SDS-PAGE and autoradiography (Fig. 1A). Autoradiographs were scanned and the ability of the mutant nsP3 MDs to remove ^32^P-MAR from MARylated PARP10^CD^ was quantified and compared to the WT set at 100% (Fig. 1B). N24A, G32E, and TM strains had no detectable activity, whereas the G32S mutant had 73.5%, the G32A mutant had 72%, and the Y114A mutant had 42% of WT activity.

MDs bind mono- and poly-ADP-ribosylated (PARylated) substrates through interactions with the terminal ADPr moiety (59–61). Therefore, we reasoned that a free PAR chain labeled at the 1″ terminus with a fluorescent probe could serve as a substrate for determining the binding affinity of the SINV nsP3 MDs for ADP-ribosylated substrates using fluorescence-based biophysical methods such as microscale thermophoresis (MST) (62). To generate a defined PAR chain fluorescently labeled at the 1″ terminus, we used enzymatically made PAR and cleaved it from modified proteins with alkaline hydrolysis that results in loss of the terminal phosphoribose moiety, generating a 5′-phosphate (63). After purifying PAR to defined length with high-performance liquid chromatography (HPLC), 1-ethyl-3-(*N*,*N*-dimethylamino)isopropyl carbodiimide (EDC) coupling was used to introduce an alkyne at the 1″ terminus of (ADPr)_18_. Cy5-azide was then conjugated to alkyne-(ADPr)_18_ via Cu(I)-catalyzed cycloaddition. Cy5-(ADPr)_18_ was incubated with 2-fold serial dilutions of SINV WT and mutant MDs, followed by MST analyses. From these assays, we determined that the nsP3 MD WT binds to Cy5-(ADPr)_18_ with a *K_D_* of 44.49 ± 2.46 μM (Fig. 1C), a value comparable to the affinity of the homologous CHIKV nsP3 MD for free ADPr (27, 43). The affinities of SINV nsP3 MD mutants for Cy5-(ADPr)_18_, were higher for Y114A (34.81 ± 4.66 μM) and TM (7.54 ± 0.65 μM), while G32A (46.39 ± 3.19 μM) resulted in no change in affinity, and all other mutations (G32S, 87.78 ± 7.71 μM; N24A 83.54 ± 6.55 μM) had reduced affinity, with G32E (849.76 ± 76.79 μM) essentially showing no ability to bind PAR which serves as a proxy for ADP-ribosylated substrates in cells (Fig. 1C).

### Effects of nsP3 MD mutations on SINV replication in mouse neuronal cells.

**FIG 2:**
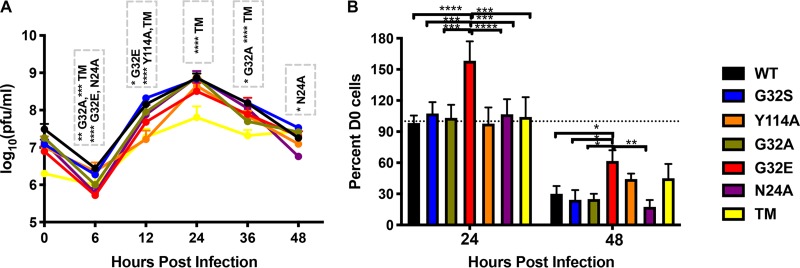
Replication of SINV WT and nsP3^MD^ mutants in mouse neuronal NSC34 cells. NSC34 cells were infected with SINV WT (TE strain) and nsP3 MD mutants N24A, G32A, G32E, G32S, Y114A, and TM (G32E/I113R/Y114N) at an MOI of 10. (A) Virus production was measured by plaque formation in Vero cells. The data are presented as means ± the SD obtained from three independent experiments *, *P* < 0.05; **, *P* < 0.01; ***, *P* < 0.001; ****, *P* < 0.0001 (TE versus nsP3 MD mutants N24A, G32A, G32E, Y114A, and TM). (B) Cell viability after infection was determined by trypan blue exclusion. The data are presented as means ± the SD obtained from three independent experiments of the numbers of viable cells compared to day 0 expressed as a percentage. *, *P* < 0.05; **, *P* < 0.01; ***, *P* < 0.001; ****, *P* < 0.0001 (G32E versus TE and nsP3 MD mutants N24A, G32A, G32S, Y114A, and TM). Significance was determined by two-way ANOVA with Tukey’s multiple-comparison test.

The nsP3 MD mutations were introduced into clones of the TE strain of SINV (5, 6), full-length viral RNA was transcribed and viruses recovered after transfection of RNA into BHK cells. All viruses could be rescued. To assess the effects of MD mutations on replication efficiency in mouse neuronal cells, NSC34 cells were infected (multiplicity of infection [MOI] of 10) with WT SINV and SINV with each of the nsP3 MD single mutations (N24A, G32A, G32E, G32S, and Y114A) or set of mutations (G32E/I113R/Y114N; TM) (Fig. 2). Peak virus production was at 24 h for all viruses (Fig. 2A). At 12 h, the amount of infectious virus released was similar to WT for G32S, G32A, and N24A, whereas G32E, Y114A, and TM had produced less virus. Infectious virus production by TM was most impaired (Fig. 2A), and sequencing of virus from 12 and 24 h showed mutation of G32E to G32S and reversion of position 113 from R to I and position 114 from N to Y. Cells infected with G32E showed less cell death compared to cells infected with other viruses at 24 and 48 h (Fig. 2B). Sequencing of viruses recovered at 12 and 24 h revealed that, in addition to the TM changes, G32E had reverted from E to WT G. Thus, altered MD function associated with mutation of G32 to E that eliminates hydrolase activity and ADPr-binding (Fig. 1) resulted in selection for reversion to WT G or to the less impaired mutant S (Fig. 1) during growth in NSC34 cells. The other mutations (N24A, G32S, G32A, and Y114A) were retained during replication in neural cells.

### Effects of nsP3^MD^ mutations on severity of SINV encephalomyelitis in mice.

**FIG 3:**
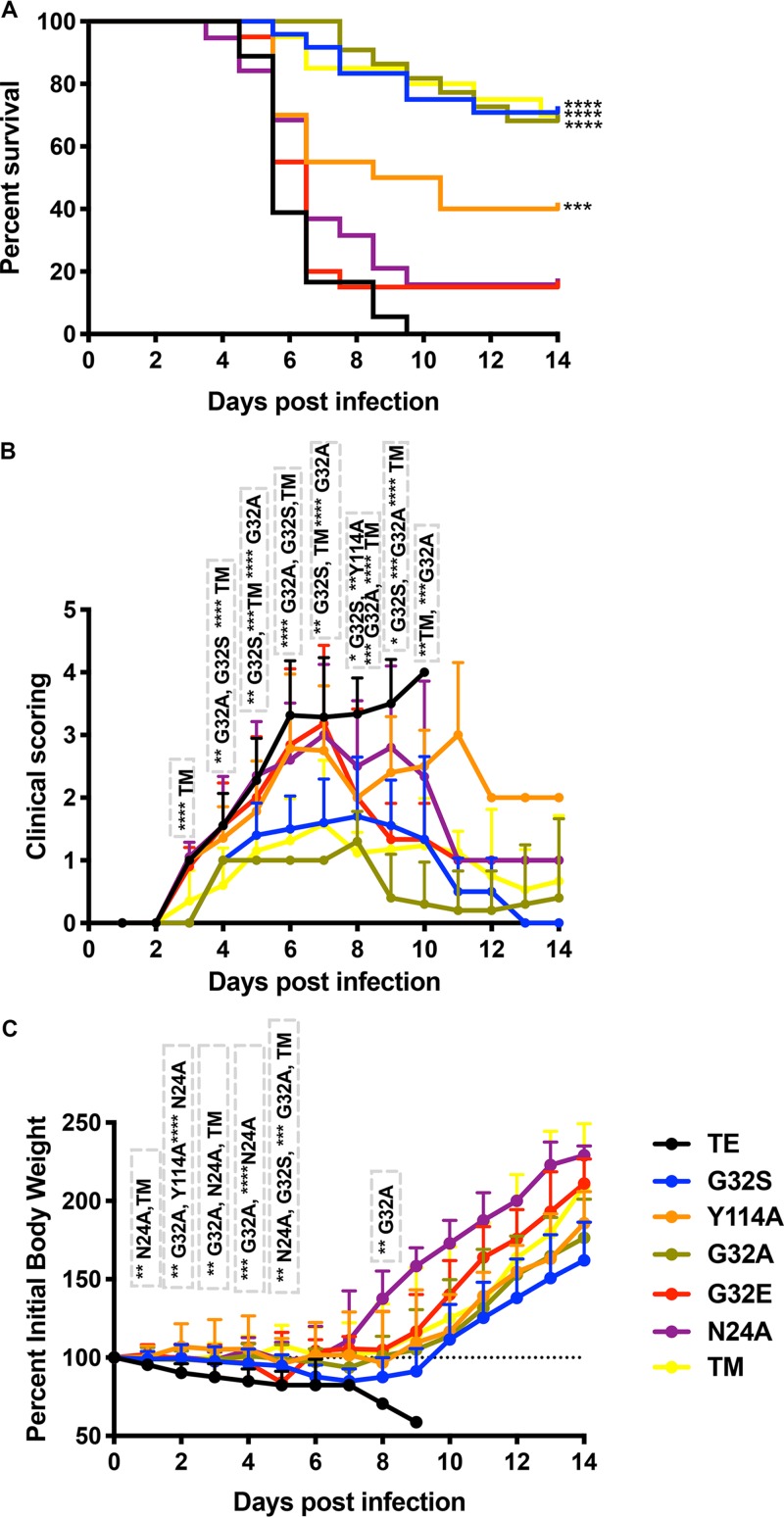
Morbidity and mortality of CD-1 mice infected with SINV WT and nsP3 MD mutants. Two-week-old CD-1 mice were inoculated intracranially with 1,000 PFU of SINV WT or nsP3 MD mutants N24A, G32A, G32E, G32S, Y114A, and TM (G32E/I11R/Y114N) and evaluated daily for 14 days. (A) Survival was assessed by Kaplan-Meier analysis and log rank Mantel Cox test. Survival was 0% for mice infected with WT TE with a mean day of death (MDOD) of 6.2; 15.8% for N24A with MDOD of 6.4, 68.2% for G32A with MDOD of 9.6, 15% for G32E with MDOD 6, 70.8% for G32S with MDOD of 8.2, 70% for TM with MDOD of 8.7, and 40% for Y114A with MDOD of 6.8. The data are from 20 to 22 mice per group of two independent experiments. ****, *P* < 0.0001 (WT versus G32S, G32A, and TM); ***, *P* < 0.001 (WT versus Y114A). (B) Signs of disease assessed using the following clinical scoring scale: 0, clinically normal; 1, ataxia and abnormal gait and tail posture; 2, hunched posture with occasional hind limb rearing (abnormal gait but normal locomotor activity); 3, severe hunched posture with limited to no locomotor activity (paralysis); and 4, death. The data are presented as the proportion of mice (18 to 20/group; 10 for G32S and G32A) showing clinical signs each day as means ± the SD. *P* values were determined by multiple *t* tests using the Holm-Sidak method. *, *P* < 0.05; **, *P* < 0.01; ***, *P* < 0.001; ****, *P* < 0.0001 (WT versus G32S, G32A, TM, or Y114A). (C) Body weight measured daily, normalized to body weight at the time of infection (dotted line) and represented as the percent body weight change. The data are presented as means ± the SD for 10 mice per group. *P* values were determined by using multiple Student *t* tests with the Holm-Sidak method. **, *P* < 0.01; ***, *P* < 0.001; ****, *P* < 0.0001 (WT versus N24A, G32A, G32S, TM, or Y114A).

The TE WT strain of SINV causes fatal encephalomyelitis in 2-week-old mice (52). To determine the role of the nsP3 MD in the pathogenesis of encephalomyelitis, we infected 2-week-old CD1 mice intracranially with 1,000 PFU of WT SINV and nsP3 MD mutants N24A, G32A, G32E, G32S, Y114A, and TM (Fig. 3). Signs of disease appeared later (day 4) in mice infected with G32A, G32S, and TM than in mice infected with WT SINV or mutants N24A, G32E, and Y114A (day 3) (Fig. 3B). Mice infected with WT virus developed ataxia and an abnormal gait with progressive paralysis, and all died by 9 days postinfection (mean day of death [MDOD] = 6.2) (Fig. 3A). Surprisingly, mice infected with G32E also developed fatal encephalomyelitis with a time course similar to that of WT virus-infected mice (MDOD = 6), as did mice infected with N24A (84% mortality; MDOD = 6.4). Disease was least severe in mice infected with G32A with 32% mortality (MDOD = 9.6, *P* < 0.0001), G32S with 29% mortality (MDOD = 8.2, *P* < 0.0001) and TM with 30% mortality (MDOD = 8.7, *P* < 0.0001) (Fig. 3A). Surviving mice in these groups recovered neurologic function by 14 days after infection (Fig. 3B). Mice infected with Y114A had an intermediate phenotype with 60% mortality (MDOD = 6.8, *P* < 0.001) (Fig. 3A), and surviving mice had persistent hind limb paralysis (Fig. 3B) despite a gain in body weight that was similar to that of G32S and G32A survivors (Fig. 3C).

To determine whether viruses reverted or developed compensatory mutations during *in vivo* CNS replication, the MDs of recovered viruses were sequenced. As observed during replication in NSC34 cells, G32E reverted to WT G and TM mutated to G32S and reverted to 113I and 114Y, while N24A retained this mutation. Thus, the two viruses with the G32E MD mutation were rapidly selected against during growth in neural cells both *in vitro* and *in vivo*. For G32E, reversion to G conferred WT virulence in mice, while selection of G32S in TM, along with reversion of position 113 to I and position 114 to Y led to virulence similar to single mutant G32S (Fig. 3A). Viruses with other MD mutations were less neurovirulent than WT but differed in whether survivors recovered neurologic function. Because G32E and TM reverted, N24A had been studied previously (55), and G32S and G32A had similar phenotypes, subsequent studies focused on comparisons between WT, G32S (decreased binding and hydrolase) and Y114A (increased binding and decreased hydrolase).

### Effects of nsP3 MD mutations on CNS virus replication and clearance.

**FIG 4:**
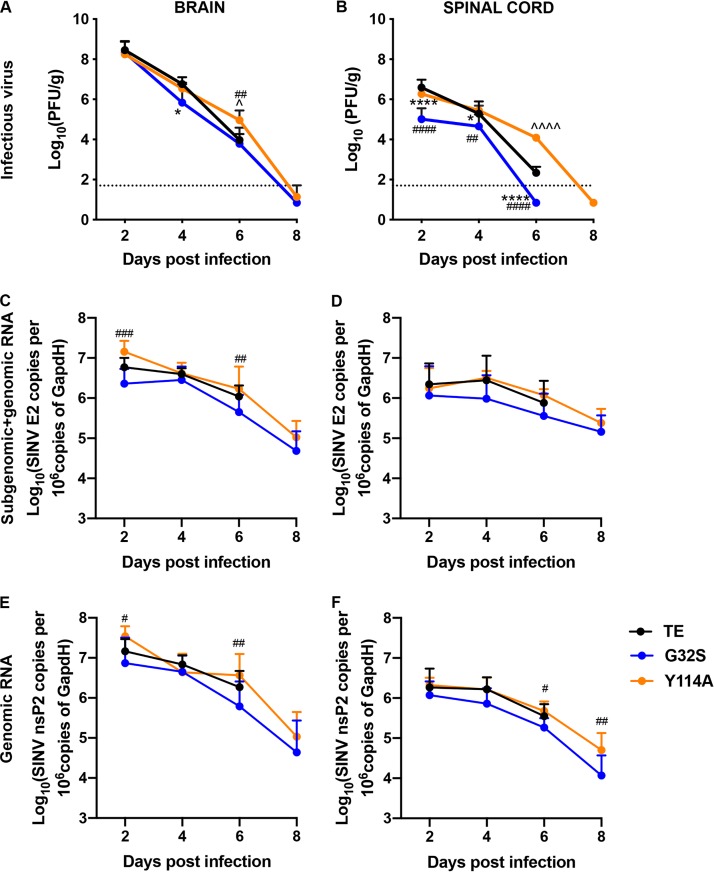
Infectious virus and SINV RNA in brains and spinal cords of mice infected with WT and nsP3 MD mutants. Two-week-old CD-1 mice were inoculated intracranially with 1,000 PFU of SINV WT or nsP3 MD mutants G32S and Y114A. Brain (A) and spinal cord (B) homogenates from four mice from each group at each time point were assayed for infectious virus by plaque assay. RNA extracted from brain and spinal cord tissues was assayed for viral subgenomic and genomic (C and D) and genomic (E and F) RNA by qRT-PCR. Data pooled from two independent experiments are presented as means ± the SD for eight mice for each time point per group. Significance was determined by two-way ANOVA with Tukey’s multiple-comparison test. *, *P* < 0.05; ****, *P* < 0.0001 (WT versus G32S). ^, *P* < 0.05; ^̂̂̂, *P* < 0.0001 (WT versus or Y114A). #, *P* < 0.05; ##, *P* < 0.01; ###, *P* < 0.001; ####, *P* < 0.0001 (G32S versus Y114A).

To determine the effect of nsP3 MD mutations on virus replication and clearance from the CNS, brain and spinal cord homogenates were assayed for SINV by plaque assay and for viral RNA by quantitative reverse transcription-PCR (qRT-PCR) (Fig. 4). In brain, all three viruses had similarly high levels of infectious virus 2 days after infection but differed in clearance (Fig. 4A). G32S was cleared more rapidly than WT (*P* < 0.05, day 4), while Y114A was cleared more slowly than WT (*P* < 0.05, day 6). In the spinal cord, the amount of virus at day 2 was lower for G32S than for the WT and Y114A (*P* < 0.0001), and the clearance of infectious virus occurred earlier (day 6). Y114A had titers similar to WT through day 4, but the amounts of infectious virus remained high at day 6 (*P* < 0.0001, WT versus Y114A) (Fig. 4B). SINV genomic and subgenomic RNAs were quantified in the brains (Fig. 4C and E) and spinal cords (Fig. 4D and F) as determined by qRT-PCR using primers specific for nsP2 (genomic; Fig. 4E and F) and E2 (subgenomic and genomic; Fig. 4C and D). The levels of viral RNA were generally lower for G32S than for the WT in both the brain and spinal cord at all times after infection, while the levels of Y114A were similar or higher than the WT. Therefore, replication of G32S in the CNS was impaired compared to the WT with rapid clearance, whereas the replication of Y114A was similar to the WT, but with impaired clearance.

### Effects of nsP3 MD mutations on expression of *Parp* mRNAs in the CNS.

**FIG 5:**
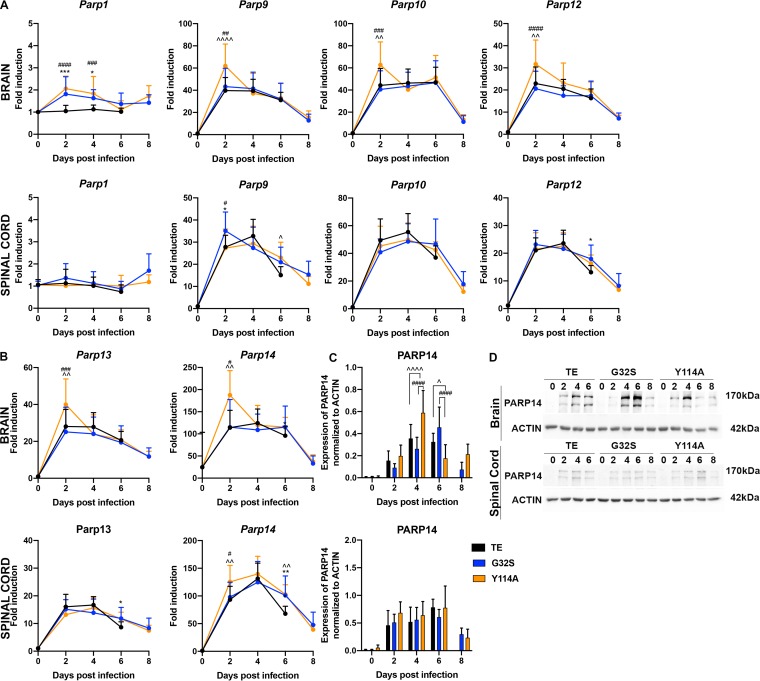
Modulation of PARP mRNA and protein expression in the CNS of mice infected with WT and nsP3 MD mutants. Two-week-old CD-1 mice were inoculated intracranially with 1,000 PFU of SINV WT (TE) or nsP3 MD mutants G32S and Y114A. RNA was extracted from brain and spinal cord tissues and the expression of *Parp1*, *Parp9*, *Parp10*, and *Parp12* (A) and of *Parp13* and *Parp14* (B) (upper panels, brain; lower panels, spinal cord) mRNAs were measured by qRT-PCR. *C_T_* values were normalized to *Gapdh*, and the fold change was calculated relative to samples from day 0 (ΔΔ*C_T_*). Data pooled from two independent experiments are presented as means ± the SD for eight mice per group. Significance was determined by two-way ANOVA with Tukey’s multiple-comparison test. *, *P* < 0.05; **, *P* < 0.01; ***, *P* < 0.001 (WT versus G32S). ^, *P* < 0.05; ^̂, *P* < 0.01; ^̂̂̂, *P* < 0.0001 (WT versus Y114A). #, *P* < 0.05; ##, *P* < 0.01; ###, *P* < 0.001; ####, *P* < 0.0001 (G32S versus Y114A). (C) Immunoblots of brain and spinal cord homogenates (20 μg of 10% [wt/vol]) probed for PARP14. Antibody against β-actin was used for loading controls. The levels of PARP14 (170-kDa band) relative to actin in the brain (upper panel) and spinal cord (lower panel) were determined using densitometry from five blots for brain and four blots for spinal cord and presented as a bar graph. Significance was determined by 2-way ANOVA with Tukey’s multiple-comparison test. ^, *P* < 0.05; ^̂̂̂, *P* < 0.0001 (WT versus Y114A). ####, *P* < 0.0001 (G32S versus Y114A). (D) Representative immunoblot images of brain (upper) and spinal cord (lower) homogenates probed for PARP14 and actin.

Several PARPs are under diversifying evolutionary pressure, are induced by IFN, and/or have demonstrated antiviral activity, suggesting that MD function may be important for countering the antiviral effects of ADP-ribosylated proteins (46, 50, 51, 64, 65). In addition, although *Parp* mRNAs are not induced by infection of NSC34 cells, PARPs are activated for ADP-ribosylation and facilitate CHIKV replication (37). To determine whether *Parp* mRNA expression was induced by CNS infection, we measured changes in levels of selected *Parp* mRNAs in brain and spinal cord by qRT-PCR (Fig. 5A and B). Expression of *Parp1* mRNA changed little, but mRNAs for *Parp9*, *-10*, *-12*, *-13*, and *-14* were increased by infection. At 2 days after infection, the brain levels of *Parp9*, *-10*, *-12*, *-13*, and *-14* mRNAs and the spinal cord levels of *Parp14* mRNA were higher in Y114A-infected mice than in either WT or G32S-infected mice. To determine whether PARP14 protein levels were increased after infection, brain and spinal cord homogenates were probed by immunoblotting (Fig. 5C and D). PARP14 protein levels increased in the CNS after infection with all three viruses with higher levels at 4 days (*P* < 0.0001 versus WT/G32S) and lower levels at 6 days (*P* < 0.05 versus WT, *P* < 0.0001 versus G32S) in the brains of Y114A-infected mice. Therefore, a subset of *Parp* mRNAs was increased after infection with generally higher levels induced by Y114A than WT or G32S infection.

### Effects of nsP3 MD mutations on IFN pathway activation in the CNS.

**FIG 6:**
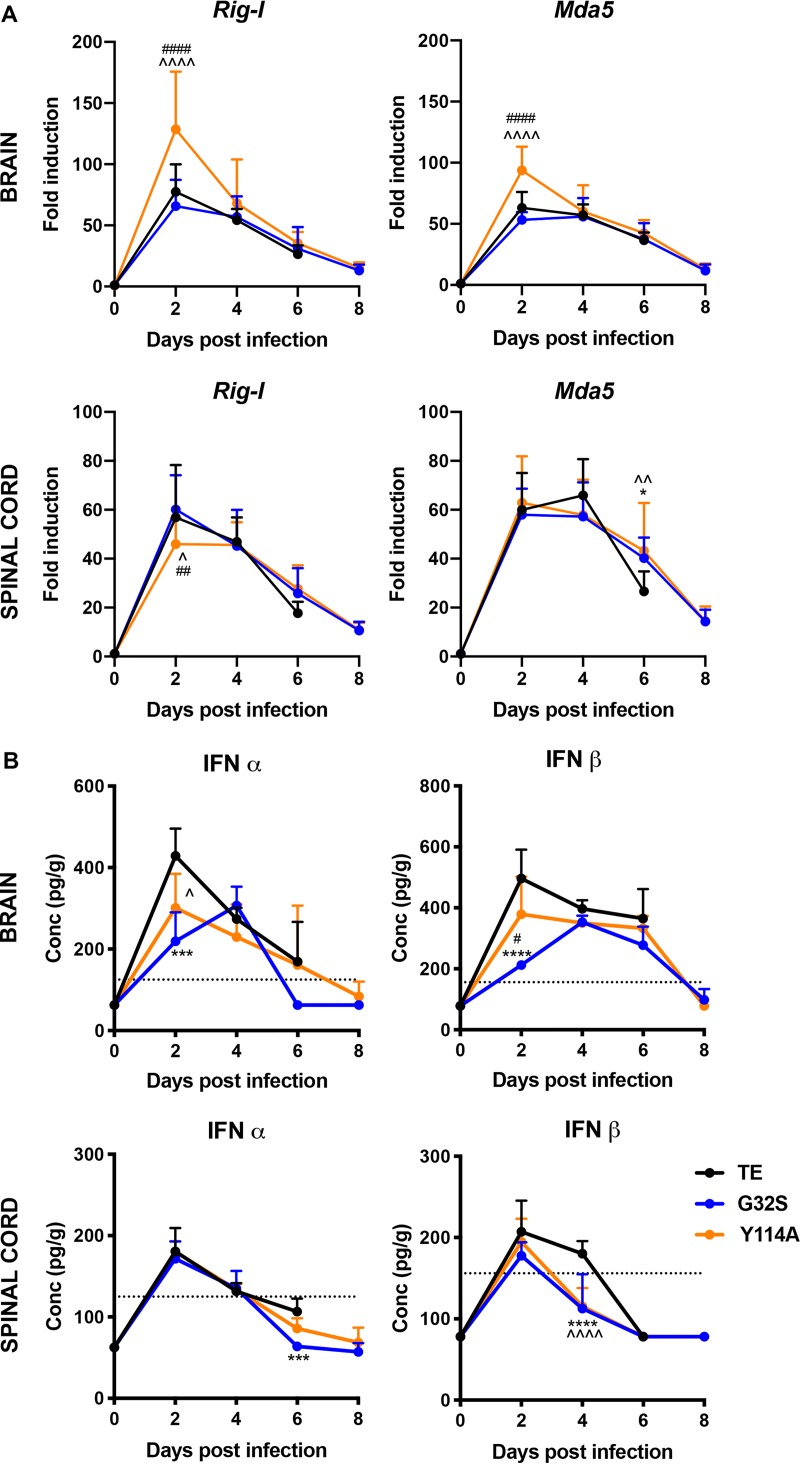
IFN signaling pathway in the CNS of the mice infected with WT and nsP3 MD mutants. Two-week-old CD-1 mice were inoculated intracranially with 1,000 PFU SINV TE or nsP3 MD mutants G32S and Y114A. (A) RNA was extracted from brain and spinal cord tissues, and the levels of *Rig-I* and *Mda5* mRNAs were measured by qRT-PCR (upper panels, brain; lower panels, spinal cord). *C_T_* values were normalized to *Gapdh*, and the fold change was calculated relative to day 0 (ΔΔ*C_T_*). Data pooled from two independent experiments are presented as means ± the SD for eight mice for each time point per group. Significance was determined by two-way ANOVA with Tukey’s multiple-comparison test. *, *P* < 0.05 (WT versus G32S). ^, *P* < 0.05; ^̂, *P* < 0.01; ^̂̂̂, *P* < 0.0001 (WT versus Y114A). ##, *P* < 0.01; ####, *P* < 0.0001 (G32S versus Y114A). (B) Brain (top) and spinal cord (bottom) homogenates were tested by EIA for IFN-α (left) and IFN-β (right). The graphs show the average concentrations of IFN in pg/g of tissues from four animals per group. The dotted line indicates the lowest assay range value in pg/ml. Significance was determined by two-way ANOVA with Tukey’s multiple-comparison test. ***, *P* < 0.001; ****, *P* < 0.0001 (WT versus G32S). ^, *P* < 0.05; ^̂̂̂, *P* < 0.0001 (WT versus Y114A). #, *P* < 0.05 (G32S versus Y114A).

Because *Parp9*, *-10*, *-12*, *-13*, and *-14* are IFN inducible (46, 66–70), the MD-mediated regulation of innate responses was examined further (Fig. 6). The expression of mRNAs for *Rig-I* and *Mda5*, important cytoplasmic sensors of viral RNAs, was analyzed in the brains and spinal cords of infected mice by qRT-PCR (Fig. 6A). *Rig-I* and *Mda5* were induced in all groups with higher levels of both mRNAs at day 2 in the brains of Y114A compared to WT- or G32S-infected mice (*P* < 0.0001), but with similar levels in the spinal cord. To compare levels of type I IFNs in the CNS after infection, the levels of alpha interferon (IFN-α) and IFN-β protein in homogenates of the brain and spinal cord were assessed by enzyme immunoassay (EIA) (Fig. 6B). WT virus induced more IFN than either Y114A or G32S (*P* < 0.05 versus Y114A, *P* < 0.001 versus G32S IFN-α day 2 brain; *P* < 0.001 versus G32S IFN-α day 6 spinal cord; *P* < 0.0001 versus G32S IFN-β day 2 and *P* < 0.0001 versus G32S/Y114A IFN-β day 4 spinal cord). Therefore, infection with WT SINV induced more brain IFN, but the expression of *Parp*s, *Rig-I*, and *Mda5* IFN-stimulated genes (ISGs) was highest in response to Y114A infection.

**FIG 7:**
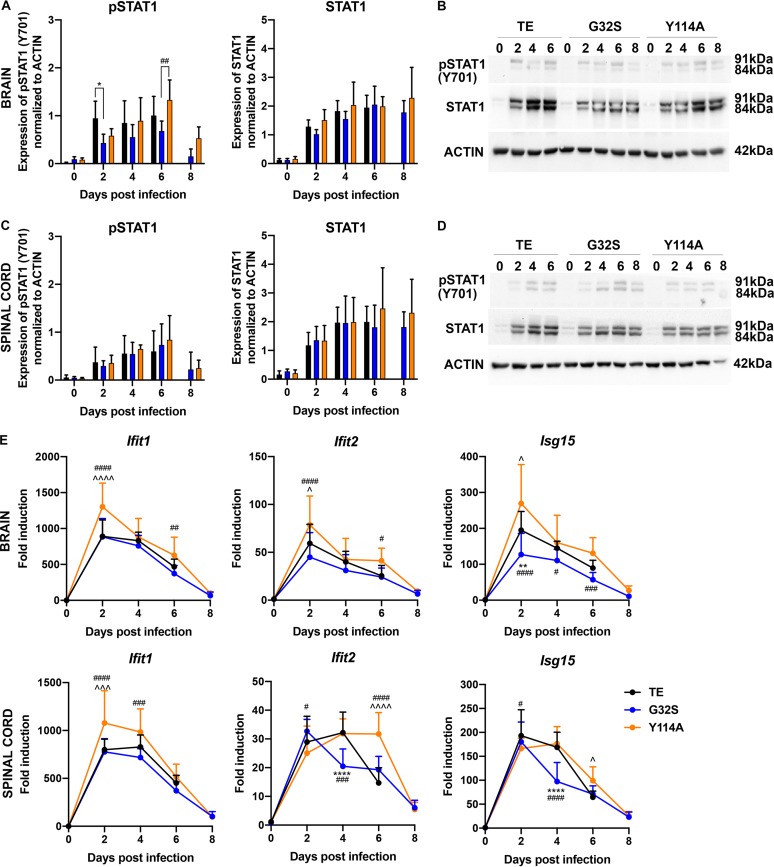
STAT1 activation and ISG expression in the CNS of mice infected with WT and nsP3 MD mutants. Two-week-old CD-1 mice were inoculated intracranially with 1,000 PFU of SINV TE or nsP3 MD mutants G32S and Y114A. Immunoblots of brain (A and B) and spinal cord (C and D) homogenates (20 μg of 10% [wt/vol]) were probed for total and phosphorylated STAT1. Antibody against β-actin was used for loading controls. The levels of pSTAT1 (Y701) and STAT1 (91- + 84-kDa band) relative to the actin in brain (A) and spinal cord (C) were quantitated using densitometry from five blots and are presented as a bar graph. Significance was determined by two-way ANOVA with Tukey’s multiple-comparison test. *, *P* < 0.05 (WT versus G32S). ##, *P* < 0.01 (G32S versus Y114A). Representative immunoblot images of brain (B) and spinal cord (D) homogenates probed for pSTAT1, STAT1, and actin. (E) RNA extracted from brain and spinal cord tissues assayed for mRNA expression of *Ifit1*, *Ifit2*, and *Isg15* by qRT-PCR. *C_T_* values were normalized to *Gapdh*, and the fold change was calculated relative to infected controls at day 0 (ΔΔ*C_T_*). Data pooled from two independent experiments are presented as means ± the SD for eight mice for each time point per group. Significance was determined by two-way ANOVA with Tukey’s multiple-comparison test. *, *P* < 0.05; **, *P* < 0.01; ***, *P* < 0.001; ****, *P* < 0.0001 (WT versus G32S). ^, *P* < 0.05; ^̂̂, *P* < 0.001; ^̂̂̂, *P* < 0.0001 (WT versus Y114A). #, *P* < 0.05; ##, *P* < 0.01; ###, *P* < 0.001; ####, *P* < 0.0001 (G32S versus Y114A).

To assess potential differences in IFN signaling, expression and phosphorylation of STAT1, an important cytoplasmic transcription factor activated by Jak kinase-mediated tyrosine phosphorylation in response to IFN (71, 72), was examined by immunoblotting brain and spinal cord homogenates (Fig. 7A to D). CNS expression of STAT1 protein was increased in response to infection by all three viruses. Activation of STAT1 (pSTAT1) in brain was evident by 2 days after infection, with lower levels for G32S-infected mice (day 2 *P* < 0.05 versus WT, day 6 *P* < 0.01 versus Y114A) (Fig. 7A to D), but no differences in the spinal cord were detected.

To further assess induction of ISGs, changes in levels of mRNAs for IFN-induced protein with tetratricopeptide repeat 1 (*Ifit1*), *Ifit2*, and *Isg15* were measured (Fig. 7E). At 2 days after infection, the levels of *Ifit1* mRNA were higher in the brains (*P* < 0.0001 versus WT/G32S) and spinal cords (*P* < 0.001 versus WT, *P* < 0.0001 versus G32S) of mice infected with Y114A. The level of *Ifit2* mRNA was also higher in the brains of Y114A-infected mice at 2 days (*P* < 0.05 versus WT, *P* < 0.0001 versus G32S) and remained at this level at 6 days in both brains (*P* < 0.05 versus WT/G32S) and spinal cords (*P* < 0.0001 versus WT/G32S). *Isg15* mRNA was higher in brains of WT- and Y114A-infected mice than G32S-infected mice throughout the infection and in the spinal cord at 4 days (*P* < 0.0001). Therefore, although IFN levels in the brain were highest with WT infection (Fig. 6B), Y114A generally induced a more vigorous and sustained ISG response than G32S or WT virus infection (Fig. 5, 6A, and 7), perhaps associated with the generally higher levels and slower clearance of viral RNA (Fig. 4).

### Effects of nsP3 MD mutations on NF-κB pathway activation in the CNS.

**FIG 8:**
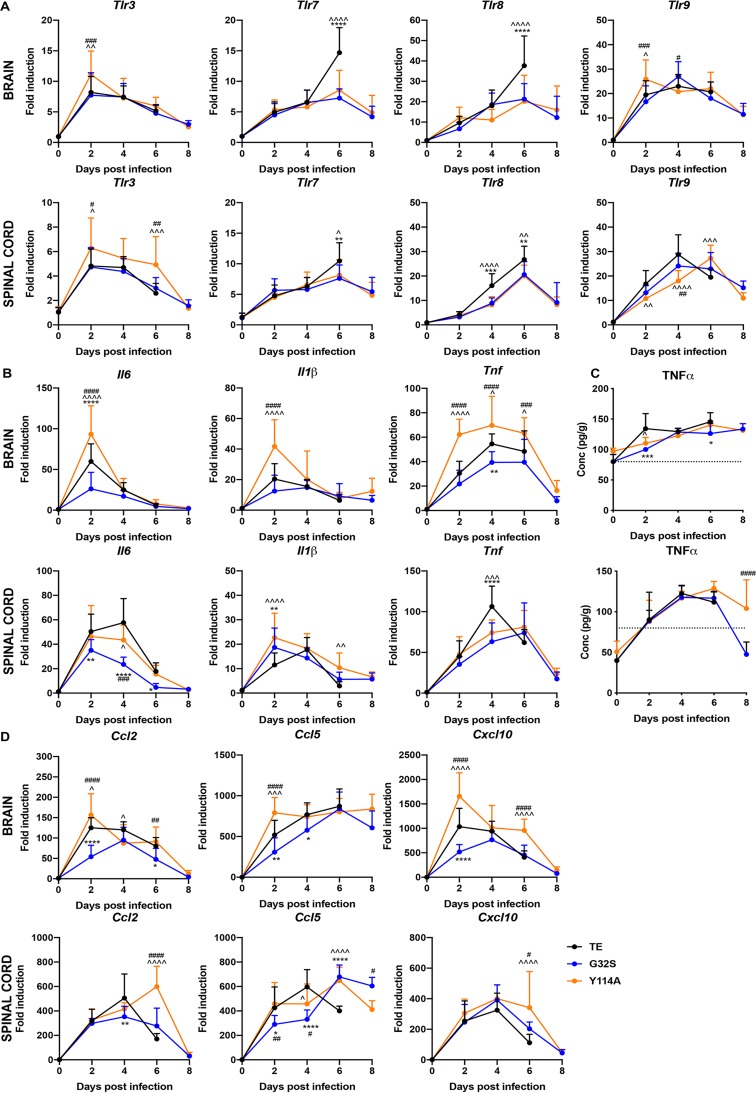
Expression of Toll-like receptors, cytokines, and chemokines in the CNS of mice infected with WT and nsP3 MD mutants. Two-week-old CD-1 mice were inoculated intracranially with 1,000 PFU of SINV TE or nsP3 MD mutants G32S and Y114A. RNA was extracted from brain and spinal cord tissues and mRNA expression of *Tlr3*, *Tlr7*, *Tlr8*, and *Tlr9* (A) and *Il6*, *Il1β*, and *Tnf* (B) was measured by qRT-PCR (upper panels, brain; lower panels, spinal cord). The *C_T_* values were normalized to *Gapdh*, and the fold change was calculated relative to day 0 (ΔΔ*C_T_*). Data pooled from two independent experiments are presented as means ± the SD for eight mice for each time point per group. Significance was determined by two-way ANOVA with Tukey’s multiple-comparison test. (C) Brain (upper panel) and spinal cord (lower panel) homogenates were tested by EIA for TNF-α. Graphs show the average concentration of TNF-α in pg/g tissue from four animals per group. The dotted line indicates the lowest assay range value in pg/ml. Significance was determined by two-way ANOVA with Tukey’s multiple-comparison test. (D) Expression of *Ccl2*, *Ccl5*, and *Cxcl10* mRNAs (upper panels, brain; lower panels, spinal cord) was measured by qRT-PCR. The *C_T_* values were normalized to *Gapdh*, and the fold change was calculated relative to day 0 (ΔΔ*C_T_*). Data pooled from two independent experiments are presented as means ± the SD for eight mice per group for each time point. Significance was determined by two-way ANOVA with Tukey’s multiple-comparison test. *, *P* < 0.05; **, *P* < 0.01; ***, *P* < 0.001; ****, *P* < 0.0001 (WT versus G32S). ^, *P* < 0.05; ^̂, *P* < 0.01; ^̂̂, *P* < 0.001; ^̂̂̂, *P* < 0.0001 (WT versus Y114A). #, *P* < 0.05; ##, *P* < 0.01; ###, *P* < 0.001; ####, *P* < 0.0001 (G32S versus Y114A).

Because NF-κB pathway activation is an important component of the innate response to viral infection that occurs in response to signaling through Toll-like receptors (TLRs), we assessed the changes in the expression of the mRNAs for endosomal TLRs 3, 7, 8, and 9 that were most relevant for responses to viral infection (Fig. 8A). All endosomal *Tlr* mRNAs were increased after infection. *Tlr3* mRNA was most rapidly induced (day 2) and more highly expressed in the brains (*P* < 0.01 versus WT, *P* < 0.001 versus G32S) and spinal cords (*P* < 0.05) of mice infected with Y114A than in mice infected with WT or G32S. At 6 days after infection, the levels of *Tlr7* and *Tlr8* in both the brain (*P* < 0.0001) and the spinal cord (*P* < 0.01) were higher in WT-infected than in Y114A- or G32S-infected mice.

The levels of mRNAs for several cytokines and chemokines dependent on NF-κB pathway signaling were examined (Fig. 8B and D). *Il1β*, *Tnf*, *Il6*, *Ccl2*, *Ccl5*, and *Cxcl10* mRNAs were all increased in the CNS after infection. In brain, levels of all innate response gene mRNAs were generally highest at 2 days in Y114A-infected mice and lowest in G32S-infected mice. In the spinal cord, WT-infected mice had higher levels of *Il6*, *Tnf*, *Ccl2*, and *Ccl5* mRNAs at 2 or 4 days after infection, while *Il1β* was highest in Y114A at 2 and 6 days (*P* < 0.01 versus G32S, *P* < 0.0001 versus Y114A). The levels of mRNAs for chemokines *Ccl2*, *Ccl5*, and *Cxcl10* remained higher at 6 days after infection in spinal cords of Y114A-infected than WT-infected mice (*P* < 0.0001) (Fig. 8B and D). The levels of tumor necrosis factor alpha (TNF-α) protein in the CNS also increased after infection and were higher in WT-infected mice at 2 days postinfection in the brain (*P* < 0.05 versus Y114A, *P* < 0.001 versus G32S) and remained high in the spinal cords of Y114A-infected mice at 8 days (*P* < 0.0001) (Fig. 8C). Therefore, Y114A-infected mice tended to have higher early levels of these cytokine and chemokine mRNAs in brain than WT mice and persistent expression in spinal cord, whereas the responses of G32S-infected mice were generally lower than WT-infected mice.

### Effects of nsP3 MD mutations on adaptive immune responses in the CNS.

**FIG 9:**
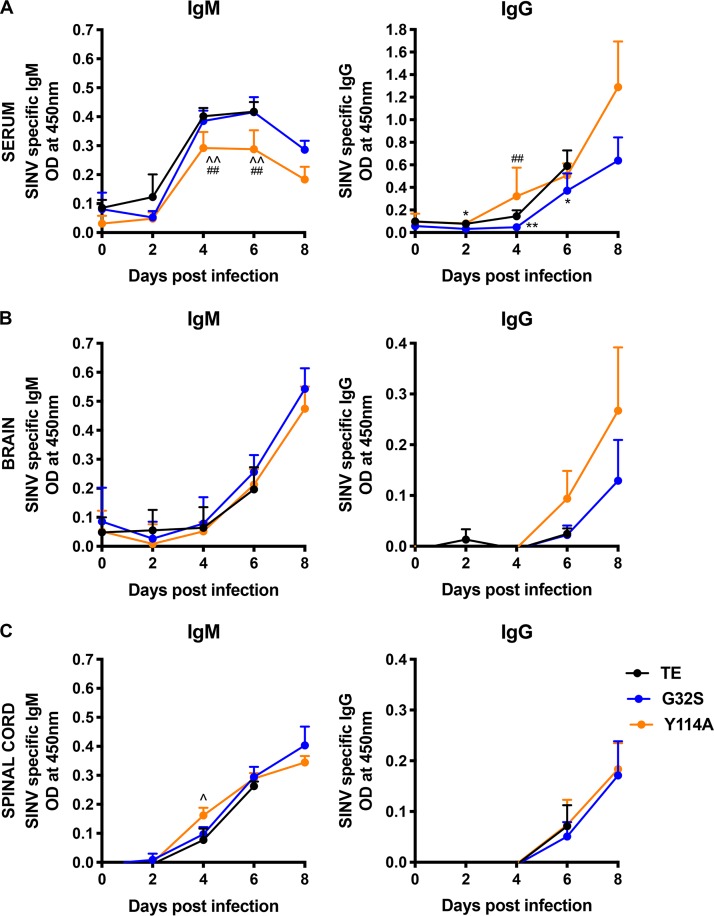
Antibody responses of mice infected with WT and nsP3 MD mutants. Two-week-old CD-1 mice were inoculated intracranially with 1,000 PFU of SINV TE and nsP3 MD mutants G32S and Y114A. Serum (A), brain (B), and spinal cord (C) homogenates were tested for SINV-specific IgM (left panels) and IgG (right panels) by EIA. The graphs show the OD of serum (1:100 dilution) and 10% (wt/vol) brain and spinal cord homogenates (1:2 dilution) from four animals per group. Significance was determined by multiple Student *t* tests using the Holm-Sidak method. *, *P* < 0.05; **, *P* < 0.01 (WT versus G32S). ^, *P* < 0.05; ^̂, *P* < 0.01 (WT versus G32S versus Y114A). ##, *P* < 0.01 (G32S versus Y114A).

To determine the effects of MD mutations on induction of the adaptive immune response, we measured the levels of SINV-specific antibodies in the serum (Fig. 9A), brains (Fig. 9B) and spinal cords (Fig. 9C) of mice infected with WT, G32S, and Y114A viruses by EIA. Serum levels of SINV-specific IgM were lower for Y114A (*P* < 0.01 days 4 and 6) than G32S and WT (Fig. 9A), whereas levels of IgG were lower in mice infected with G32S (*P* < 0.01, days 4 and 6) (Fig. 9A). Because the antibody levels in the CNS reflect entry of antibody-secreting cells into the brain and spinal cord and are most important for virus clearance and recovery (73, 74), we also measured SINV-specific antibodies in CNS tissue homogenates. In both the brain (Fig. 9B) and the spinal cord (Fig. 9C) the IgM and IgG levels were similar. These data suggest that MD mutations had little effect on induction of the antibody response to SINV.

**FIG 10:**
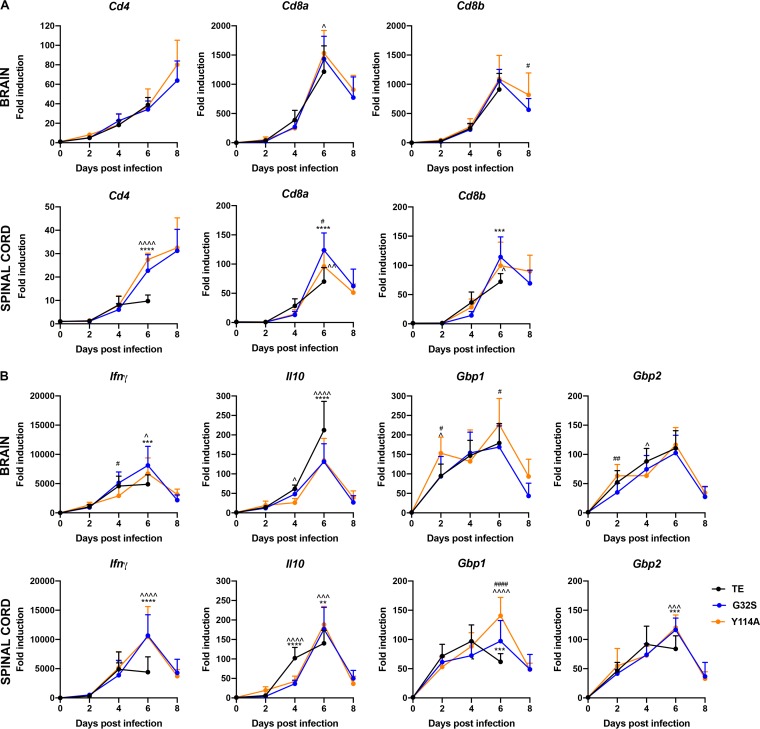
Cellular immune response genes expressed by mice infected with WT and nsP3 MD mutants. Expression of *Ifnγ*, *Il10*, and IFN-γ-induced ISG mRNAs in the CNS of mice infected with WT and nsP3 MD mutants. Two-week-old CD-1 mice were inoculated intracranially with 1,000 PFU of SINV TE or nsP3 MD mutants G32S and Y114A. RNA was extracted from brain and spinal cord tissues, and the mRNAs for *Cd4*, *Cd8a*, and *Cd8b* (A) and *Ifnγ*, *Il10*, *Gbp1*, and *Gbp2* (B) were measured by qRT-PCR (upper panels, brain; lower panels, spinal cord). The *C_T_* values were normalized to *Gapdh*, and the fold change was calculated relative to day 0 (ΔΔ*C_T_*). Data pooled from two independent experiments are presented as means ± the SD for eight mice for each time point per group. Significance was determined by two-way ANOVA with Tukey’s multiple-comparison test. *, *P* < 0.05; **, *P* < 0.01; ***, *P* < 0.001; ****, *P* < 0.0001 (WT versus G32S). ^, *P* < 0.05; ^̂, *P* < 0.01; ^̂̂, *P* < 0.001; ^̂̂̂, *P* < 0.0001 (WT versus Y114A). #, *P* < 0.05; ##, *P* < 0.01; ####, *P* < 0.0001 (G32S versus Y114A).

Because CD4 and CD8 T cells also play an important role in adaptive immune responses, we assessed changes in the CNS levels of *Cd4*, *Cd8a*, and *Cd8b* mRNAs after infection as indicators of T cell infiltration and retention (Fig. 10A). Expression of *Cd4* mRNA steadily increased, while *Cd8a* and *Cd8b* increased for 6 days and then decreased at 8 days in surviving G32S- and Y114A-infected mice. The levels of *Cd4*, *Cd8a*, and *Cd8b* mRNAs in the brain were similar in the three groups, but in the spinal cord at 6 days the mRNA levels of *Cd4* (*P* < 0.0001), *CD8a* (*P* < 0.0001), and *CD8b* (*P* < 0.001) were lowest in WT-infected mice (Fig. 10A).

Because the primary T cell effector of SINV clearance is IFN-γ with interleukin-10 (IL-10) as an important regulator (74–76), these cytokine mRNAs were measured as additional indicators of T cell responses in the CNS (Fig. 10B). *Ifnγ* mRNA was highly induced in all animals and, at day 6, the level was lower in the brains and spinal cords of mice infected with WT virus than in G32S (*P* < 0.001, brain; *P* < 0.0001 spinal cord)- or Y114A (*P* < 0.05, brain; *P* < 0.0001, spinal cord)-infected mice (Fig. 10B). The expression of IFN-γ-induced ISGs guanylate binding protein 1 (*Gbp1*) and *Gbp2* mRNAs also increased in the brains and spinal cords of all mice. In the spinal cord the levels of *Gbp1* and *Gbp2* mRNAs at day 6 were lowest for WT-infected mice (*Gbp1*, *P* < 0.0001; *Gbp2*, *P* < 0.001). The mRNA for regulatory cytokine *Il10* increased after infection, with the highest levels in the brains of WT-infected mice at 6 days (*P* < 0.0001) and in spinal cords at 4 days (*P* < 0.0001) (Fig. 10B). These data suggest that SINV with MD mutations induce a more vigorous IFN-γ-producing T cell response to infection than WT SINV, particularly in the spinal cord.

## DISCUSSION

Viral nonstructural protein MDs possess both MAR hydrolase and ADPr-binding activities (43–45) and are important determinants of neurovirulence, but how pathogenesis of alphavirus encephalomyelitis is affected by alterations in the ADPr-binding and hydrolase functions of the nsP3 MD has not been evaluated. Previous studies of CHIKV nsP3 MD mutants showed that these activities are critical for different aspects of alphavirus replication in neuronal cells (37, 43, 55). If both functions are impaired, initiation of infection is inefficient, and little if any virus is produced. If there is better binding but diminished hydrolase activity, replication is initiated more quickly, but the amplification of replication complexes is impaired (37). To determine how altered MD functions affect the pathogenesis of encephalomyelitis, we introduced mutations similar to those studied in CHIKV into SINV, a better model system for analysis of CNS alphavirus infection in mice, and showed that the effects on ADPr-binding and hydrolase activities were similar to those observed for CHIKV.

*In vitro* replication of SINV in neural cells and *in vivo* replication in the CNS of mice were severely impaired by mutations that eliminate ADPr-binding and hydrolase activities (G32E) with reversion to WT (G) or selection of a less compromising change (S) during replication. SINVs with MDs deficient in both binding and hydrolase activities (G32S and G32A) or with hydrolase deficiency combined with better binding (Y114A) were less virulent than WT virus in mice but displayed different phenotypes. G32S replicated less well in both the brain and the spinal cord, induced similar innate immune responses, and caused less severe disease, with 71% survival and full recovery of survivors. Y114A replicated better than WT and induced higher expression of IFN-stimulated and NF-κB-induced genes with similar antibody responses but was cleared more slowly from the spinal cord, with 40% survival and persistent neurologic deficits in survivors. Therefore, MD function was important for neural cell replication both *in vitro* and *in vivo* and determined the outcome from alphavirus encephalomyelitis in mice.

Alanine substitution at residue 10 eliminates alphavirus MD ADPr-binding and is not tolerated by CHIKV (D10) or by SINV (N10), and D is present at this position in the encephalitic New World alphaviruses VEEV, EEEV, and WEEV (43, 55). Mutation of this critical binding residue leads to a failure to synthesize viral RNA in neural cells, so that any virus recovered had either reverted to D, T, or N or acquired a compensatory mutation at position 31 (E to G) (37, 55). The hydrolase loop formed by residues 24 to 33 also participates in ADPr-binding (necessary for enzyme function), but enzymatic activity *per se* is less critical for initiating alphavirus replication (37). The amino acid at position 24 (N) is conserved in all viral MDs examined (50, 51), and mutation of N24 to A eliminated hydrolase activity (Fig. 1) and impairs shutoff host protein synthesis in NIH 3T3 cells (56) but did not affect replication in NSC34 cells (Fig. 2A). However, mortality in 2-week-old mice was reduced from 100 to 84% in the present study (Fig. 3A) and to 40% in our previous study (55) and is associated with more rapid virus clearance. The reasons for differences in mortality between the two studies are not known but may be due to differences in genetic background or microbiome composition of outbred CD-1 mice. The phenotype was similar to that observed with mutation at the comparable position of the SARS-CoV MD (N41A/N1040A) that does not affect replication in Vero or Calu-3 2B4 epithelial cells but decreases lung virus replication and improves survival of mice after respiratory infection. These results were postulated to be linked to MD regulation of innate response genes with higher lung levels of IFN, ISG, IL-6 and TNF mRNAs at 24 h, but not 72 h, after infection (40). The specific mechanisms for these effects have yet to be identified.

The amino acid at position 32 is a critical determinant of binding, as well as enzymatic, activity, and G is highly conserved for all viral MDs, including the encephalitic alphaviruses VEEV, WEEV, and EEEV (51). E at this position eliminates both binding and hydrolase activities (Fig. 1) (43), and SINV G32E MD mutants reverted during replication in NSC34 cells and in the nervous systems of mice. Mutation of G32 to S or A partially preserved ADPr-binding, with hydrolase activity decreased 26.5% for G32S and 28% for G32A (Fig. 1). These viruses replicated less well in NSC34 cells than did the WT (Fig. 2) and induced less severe neurologic disease with reduced mortality in mice (Fig. 3). Replication of G32S in the CNS was lower than that of the WT, with the most striking difference in the spinal cord (Fig. 4). We postulate that decreased virus production is due to less efficient initiation and amplification of replication in neurons, as observed *in vitro* for CHIKV G32S (37), resulting in decreased spread of virus to the spinal cord. Therefore, efficient replication complex formation and amplification in neurons likely requires binding to one or more yet to be identified ADP-ribosylated proteins.

The affinity of MD binding to ADPr is determined in part by the amino acid at position 114 in the ADPr-binding pocket (50). The amino acid at 114 is more variable in alphaviruses than the other residues studied with a Y in EEEV and WEEV, as well as SINV and CHIKV, but F in VEEV. The Y114A substitution in SINV increases binding while decreasing hydrolase activity (Fig. 1) (43) and thus provides an opportunity to independently examine the role of MD hydrolase activity in pathogenesis. As for CHIKV (37, 43), SINV Y114A grew more slowly in NSC34 cells (Fig. 2) and was less virulent in mice (Fig. 3). However, Y114A replicated as well or better than WT SINV in the CNS, was cleared more slowly from both brain and spinal cord, and resulted in permanent neurologic damage in survivors (Fig. 4). Initial control of SINV replication in the CNS is dependent on the innate immune response, particularly local production of type I IFN (77–79). Because viral MDs can influence both the induction of and the response to innate immune effectors (40, 65, 80, 81), we assessed multiple parameters of the immune response to SINV in the CNSs of infected mice. Mice infected with Y114A in general had similar CNS levels of IFN-α and IFN-β and greater induction of ISGs *Parp9*, *-10*, *-12*, *-13*, and *-14*; *Ifit1* and -*2*; and *Isg15* (Fig. 5 and 7) and innate response genes *Tlr3*, *Il1β*, *Il6*, *Tnf*, *Ccl2*, *Ccl5*, and *Cxcl10* (Fig. 8) compared to WT-infected mice. Because these same parameters tended to be lower in G32S-infected mice where viral RNA loads were also lower than WT, we postulate that MD-determined levels of virus replication in the CNS have a more important role in regulating innate responses than direct MD regulation of these responses.

Surviving Y114A-infected animals had persistent hind limb paralysis associated with delayed virus clearance from the spinal cord (Fig. 3B and 4D). SINV clearance is mediated by the adaptive immune response with the combined effects of antibody and IFN-γ (74, 75, 82, 83). Although production of serum IgM was decreased in Y114A-infected animals compared to WT and G32S-infected mice, levels of SINV-specific IgM and IgG, as well as *Ifnγ* mRNA, in the brains and spinal cords were similar or higher (Fig. 9 and 10). Therefore, defects in clearance could not be ascribed to failure to induce an adaptive immune response. Because the mechanisms of antibody-mediated and IFN-γ-mediated clearance of virus from neurons are not known and MD hydrolase activity affects amplification of replication complexes, shutoff of host protein synthesis and translation of structural proteins (37), it is possible that MD interactions with intracellular mechanisms of virus clearance are affected by the mutations studied.

In summary, these studies have shown that MD function is an important determinant of alphavirus replication in the CNS, manifestations of encephalomyelitis, virus clearance, and recovery.

## MATERIALS AND METHODS

### Viruses, mutagenesis, and sequencing.

The nsP3 MD mutants were generated in the full-length double subgenomic clone of SINV TE (84) using a QuikChange site-directed mutagenesis kit (Agilent Technologies). The single mutations introduced were glutamine to alanine at position 24 (N24A), glycine to serine, alanine, or glutamate at 32 in the hydrolase loop (G32S, -A, or -E) and tyrosine to alanine at 114 in the binding site (Y114A). A triple mutant (TM) designed to decrease hydrolase activity without decreasing binding (glycine to glutamate at position 32, isoleucine to arginine at position 113, and tyrosine to asparagine at position 114; G32E/I113R/Y114N) was also constructed (43). Clones were sequenced using the primer TE 4101 F 5′-ACCATGGCGCCGTCATAC-3′. Viral RNAs were transcribed by using a mMESSAGE mMACHINE high-yield capped RNA transcription kit (Invitrogen) and transfected into BHK-21 cells using Lipofectamine 2000 (Invitrogen). The rescued virus was passaged once in BHK-21 cells, and viral RNAs were purified from virus released into the cell culture media using a QIAamp viral RNA minikit (Qiagen), amplified by RT-PCR using the primers TE 3437 F 5′-GCTAGCTGGGAAGGGCAC-3′ and TE 5747 R 5′-GTATTCAGTCCTCCTGCTC-3′, and products were sequenced to confirm the mutations in the virus. Viral stocks were grown and assayed by plaque formation in BHK-21 cells.

### Plasmids and protein purification.

For protein purification, the PARP10 catalytic domain (PARP10^CD^, residues 818 to 1025), SINV WT, and mutant nsP3 MDs (residues 3 to 161) were cloned into a pBAT4-derived vector containing a 6×His-SUMO tag. Constructs were transformed into either DE3 (nsP3 MDs) or DE3 Rosetta (PARP10^CD^) E. coli cells, and resulting colonies were cultured in Luria broth containing 0.03 mg/ml of ampicillin and 0.035 mg/ml of chloramphenicol (DE3 Rosetta only) to an optical density (OD) of 0.5 at 37°C. Cultures were then induced with 0.3 mM IPTG (isopropyl-β-d-thiogalactopyranoside) and grown overnight at 16°C. The cells were harvested, sonicated in binding buffer (25 mM HEPES [pH 7.0], 500 mM NaCl, 20 mM imidazole [pH 7.4], 10% glycerol, 10 mM β-mercaptoethanol, 1× SigmaFast protease inhibitor), and cleared by centrifugation. The lysate was applied to a 5-ml HiTrap Talon Crude FF column (GE), washed with 10 column volumes of binding buffer, and eluted with 250 mM imidazole in binding buffer. The eluent was desalted on a 5-ml HiTrap desalting column (GE) into binding buffer, and the 6×His-SUMO tag was cleaved by incubation with 6×His-SENP SUMO protease for 1 h at 37°C at a 1:50 enzyme/substrate ratio. Cleaved proteins were purified further by reverse IMAC on a 1 ml HisTrap Crude FF column (GE) and gel filtration chromatography on a HiLoad 16/600 Superdex 200-pg column (GE) into storage buffer (20 mM Tris [pH 7.0], 200 mM NaCl, 5% glycerol, 10 mM β-mercaptoethanol). Aliquots were snap-frozen in liquid nitrogen and stored at –80°C. All chromatography was performed on a Bio-Rad NGC Quest 10 system.

### PARP10^CD^ demodification assay.

For each reaction 1 μg of PARP10^CD^ was automodifed with 0.5 μCi of ^32^P NAD^+^ (Perkin-Elmer) for 30 min at 30°C in automodification buffer (20 mM Tris-HCl [pH 7.5], 50 mM NaCl, 5 mM MgCl_2_, 10 mM β-mercaptoethanol). Excess ^32^P NAD^+^ was removed by desalting by gravity flow in a Micro Bio-Spin column (Bio-Rad) into demodification buffer (25 mM Tris-HCl [pH 7.0], 200 mM NaCl, 10 mM β-mercaptoethanol). MARylated PARP10^CD^ was then incubated with equimolar amounts of MD proteins for 1 h at 37°C. Reactions were stopped with SDS-PAGE running buffer, and samples were subjected to SDS-PAGE on a 14% Tris-glycine gel (Invitrogen). Total protein levels were analyzed by staining with SimplyBlue Safe stain (Life Technologies), and ^32^P signal was visualized by autoradiography.

### Reagents.

EDC, imidazole, MES, sodium chloride, copper (II) sulfate, (+) sodium l-ascorbate, and THPTA were purchased from Sigma-Aldrich.

### Cy5 labeling.

Cy5-labeled PAR was synthesized as described for 5′-phosphorylated RNA with modifications (85) in a 30-μl reaction mixture containing 2 nmol of PAR, 0.1 M EDC, and 0.1 M imidazole (pH 6.0) in MES (0.25 M [pH 6.0]) at 25°C for 90 min. Sodium acetate (pH 5.2) was added to a final concentration of 0.1 M and the reaction was precipitated with 70% (vol/vol) ethanol by incubation at –80°C for 5 min and centrifugation at 4°C at 21,100 × *g* for 30 min. The pellet was washed twice with ice-cold 70% ethanol and then resuspended in 27 μl of 2.5 M 2-[2-(2-propinyloxy)ethoxy]ethylamine (TCI America) and incubated at 50°C for 1 h. Sodium acetate (pH 5.2) was added to a final concentration of 0.1 M, and the reaction was precipitated with 70% (vol/vol) ethanol by incubating at –80°C for 5 min and centrifugation at 4°C at 21,100 × *g* for 30 min. The pellet was washed twice with ice-cold 70% ethanol and then resuspended in 20 μl of a solution containing 0.2 M NaCl, 10 mM sodium ascorbate, 2 mM THPTA, 1 mM CuSO_4_, and 100 μM Cy5-azide (Sigma-Aldrich), followed by incubation at 37°C for 2 h.

### HPLC purification.

An Agilent Infinity II HPLC apparatus was loaded with 0.1 M TEAA (pH 7.5) and acetonitrile into the appropriate solvent ports. The detector was set to record 258 and 650 nm. An Infinity Poroshell 120 EC-C18 (Agilent, catalog no. 693970-902T) was connected and equilibrated with 95% TEAA (pH 7.5) and 5% acetonitrile. The crude labeling reaction was injected, and reaction components were separated with a gradient of (0 to 10 min, 5% acetonitrile; 10 to 40 min, 5 to 40% acetonitrile). Fractions (0.25 ml) were automatically collected during the entire run. Fractions of interest were combined, flash frozen in liquid nitrogen, and then lyophilized. The Cy5-labeled PAR was resuspended with 30 μl of mQ water. A UV-Vis spectrum was collected to estimate [PAR] and [Cy5] using the following equation: [PAR] = [(*A*_258_) cm^−1^]/[(19 × 13,500) cm^−1^ M^−1^)], [Cy5] = [(*A*_650_) cm^−1^]/[(250,000) cm^−1^ M^−1^)].

### Microscale thermophoresis.

MST analyses were conducted as previously described (86) with minor adjustments. Briefly, defined-length PAR prepared as described previously (86) was labeled on the 1″ terminus with Cy5 (10 nM) and incubated with 2-fold serial dilutions (diluted down from 0.5 to 1 mM stock solution to 15 to 30 nM) of SINV WT and mutant MDs. MST was measured using a Monolith NT.115 (NanoTemper) at 80% excitation power and 20% MST power. Values for the mean normalized fluorescence ± the standard deviation (SD) were determined, and the dissociation constants (*K_D_*) were calculated using MO Affinity Analysis software.

### Cell culture and quantification of infectious virus.

NSC34 mouse neuronal cells (87, 88) and BHK-21 cells were grown in Dulbecco modified Eagle medium (DMEM) supplemented with 10% heat-inactivated fetal bovine serum (FBS), l-glutamine (2 mM), penicillin (100 U/ml), and streptomycin (100 μg/ml) (Gibco/Life Technologies) at 37°C in a 5% CO_2_ incubator. NSC34 cells were infected at an MOI of 10 in DMEM with 1% FBS, followed by incubation at 37°C. Virus production was measured by plaque assay on BHK-21 cells. Cell viability was determined by trypan blue exclusion.

### Animals and infection.

Female CD-1 mice with 10-day-old litters were purchased from Charles River Laboratories (Wilmington, MA) and housed in a specific-pathogen-free animal facility at the Johns Hopkins School of Public Health. At 14 days of age, mice were lightly anesthetized with isoflurane and infected intracranially with 10^3^ PFU of virus diluted in 10 μl of PBS. All pups within a litter were infected with the same virus. Mice were weighed and monitored for signs of disease twice daily for 14 days. The following clinical number scoring system was used: 0, clinically normal; 1, ataxia and abnormal gait and tail posture; 2, hunched posture with occasional hind limb rearing (abnormal gait but normal locomotor activity); 3, severe hunched posture with limited to no locomotor activity (hind limb paralysis); and 4, death. For tissue collections, mice were anesthetized with an overdose of isoflurane, bled via cardiac puncture, and perfused with ice-cold PBS. Brains and spinal cords were collected and used fresh or snap-frozen and stored at –80°C.

To assess the virus replication in the CNS, left brain hemispheres (20% [wt/vol]) or whole spinal cords (10% [wt/vol]) from infected mice were homogenized in Lysing Matrix A tubes at 6.0 M/s for 40 s using a FastPrep-24 homogenizer (MP Biomedicals) and clarified by centrifugation at 13,200 rpm for 15 min at 4°C. The amount of infectious virus in clarified homogenates was determined by plaque formation on BHK-21 cells. The data are plotted as mean log_10_ values of PFU per g of tissue, and samples with no detectable plaques at the lowest dilution tested were assigned a value of half the limit of detection.

All experiments were performed according to protocols approved by the Johns Hopkins University Animal Care and Use Committee and carried out with strict adherence to the National Institutes of Health *Guide for the Care and Use of Laboratory Animals* and the U.S. Public Health Service Policy on Humane Care and Use of Laboratory Animals.

### RNA isolation from tissue homogenates and quantification by qRT-PCR.

Right brain hemispheres or whole spinal cords from eight animals per group per time point (from two independent experiments) were homogenized in 1 ml of Qiazol reagent in lysing matrix D tubes at 6.0 M/s for 40 s using a FastPrep-24 homogenizer (MP Biomedicals), and RNA was isolated using an RNeasy Lipid Mini RNA isolation kit (Qiagen). Briefly, cDNA was synthesized with random primers using a high-capacity cDNA reverse transcription kit (Life Technologies), and qRT-PCR was performed using TaqMan gene expression arrays (Integrated DNA Technologies), and Universal PCR Master mix (Applied Biosystems). The TaqMan gene expression arrays used were*: Cd4*, *Cd8a*, *Cd8b*, *Ifnγ*, *Il1β*, *Il6*, *Tnf, Ccl2*, *Ccl5*, *Cxcl10*, *Tlr3*, *Tlr7*, *Tlr8*, *Tlr9*, *Rig-I*, *Mda5*, *Gbp1*, *Gbp2*, *Ifit1*, *Ifit2*, *Isg15*, *Parp1*, *Parp9*, *Parp10*, *Parp12*, *Parp13*, and *Parp14*. The ΔΔ*C_T_* method was used to determine relative gene expression using day 0 samples and rodent *Gapdh* for normalization.

SINV subgenomic plus genomic RNA was quantified by qRT-PCR using TaqMan probe (SINV E2 8760 5′-6-carboxyfluorescein [FAM]-CGCATACAGACTTCCGCCCAGT-6-carboxytetramethylrhodamine [TAMRA]-3′(Applied Biosystems) and primers to the SINV E2 gene (SV 8732F-5′-TGGGACGAAGCGGACGATAA-3′; SV 8805R-5′-CTGCTCCGCTTTGGTCGTAT-3′). SINV genomic RNA was quantified using TaqMan probe (SINV nsP 3317 5′-6-carboxyfluorescein (FAM)-CCATTGCCGCCGAACTCTCCC-6-carboxytetramethylrhodamine [TAMRA]-3′) and primers to the SINV nsp2 gene (SV 3374F 5′-CCGCAAGTATGGGTACGATCA-3′; SV 3454R 5′-GTGCCCTTCCCAGCTAGCT-3′). Numbers of RNA copies were determined using a standard curve made from 10-fold dilutions of a pCRII-TOPO plasmid containing the SINV genomic or subgenomic region and normalized to endogenous rodent *Gapdh*. The data are plotted as SINV RNA copies/10^6^ copies of *Gapdh*.

### Enzyme immunoassays.

SINV-specific antibody in serum and 10% (wt/vol) brain and spinal cord homogenates was measured using an in-house EIA (89). Maxisorp 96-well plates (Thermo Scientific Nunc) were coated with 10^6^ PFU/well of SINV strain TE in 50 mM NaHCO_3_ (pH 9.6) at 4°C overnight. The wells were blocked with 10% FBS in PBS containing 0.05% Tween 20 (PBST) for 2 h at 37°C. Samples diluted (1:100 for serum and 1:2 for homogenates) in blocking buffer were added, followed by incubation overnight at 4°C. Bound antibodies were detected using horseradish peroxidase (HRP)-conjugated goat anti-mouse IgM or IgG (Southern Biotech) diluted 1:1,000 in PBST plus 10% FBS for 2 h at room temperature and developed using a BD OptEIA TMB substrate reagent kit with 2 M H_2_SO_4_ as the stop solution. Plates were read at 450 nm, and the optical density (OD) values from control wells were subtracted from the OD values for infected mice.

IFN-α, IFN-β, and TNF-α levels in brain and spinal cord homogenates (10% wt/vol) were measured using commercial ELISA kits (PBL Assay Science; Invitrogen) according to the manufacturer’s instructions. Samples from four mice per group per time point were tested in duplicate, and data are presented as pg per ml. The assay range was 12.5 to 400 pg/ml for IFN-α, 15.6 to 1,000 pg/ml for IFN-β, and 8 to 1,000 pg/ml for TNF-α.

### Immunoblot analyses.

Total protein in 10% (wt/vol) brain and spinal cord homogenates was quantified with the DC assay (Bio-Rad), and 20 μg was used for SDS-PAGE on a 10% polyacrylamide gel. The samples were electrophoresed and transferred to nitrocellulose membranes. Membranes were incubated overnight at 4°C with antibodies to STAT1, phospho-STAT1 (Cell Signaling Technology), PARP14 (kindly provided by Mark Boothby, Vanderbilt University) (90) and β-actin (Millipore) diluted in 5% BSA. The secondary antibodies were HRP-conjugated anti-rabbit or anti-mouse IgG (Cell Signaling) diluted 1:10,000 in 2% nonfat dry milk and incubated for 1 h at room temperature. Membranes were developed using an Amersham ECL Plus Western blot developing kit (GE Healthcare), and densitometric analysis was carried out using ImageJ software.

### Statistical analysis.

Survival was assessed using Kaplan-Meier curves and a log-rank (Mantel-Cox) test. Time course studies were analyzed using two-way analysis of variance (ANOVA) with Tukey’s multiple-comparison posttest to compare the groups infected with TE, G32S, and Y114A 0 to 6 days after infection. Two-way ANOVA with Bonferroni’s posttest was used to compare surviving G32S and Y114A groups at 8 days. Differences in a single group were determined using one-way ANOVA with Dunnett’s multiple-comparison test. Differences between groups at a single time point were determined using an unpaired two-tailed Student *t* test with a 95% confidence interval. The results are expressed as means ± the SD. Statistical analyses were conducted using Prism 8 (GraphPad).

## References

[1] Sane J, Kurkela S, Desdouits M, Kalimo H, Mazalrey S, Lokki ML, Vaheri A, Helve T, Tornwall J, Huerre M, Butler-Browne G, Ceccaldi PE, Gessain A, Vapalahti O 2012 Prolonged myalgia in Sindbis virus infection: case description and *in vitro* infection of myotubes and myoblasts. J Infect Dis 206:407–414. doi:10.1093/infdis/jis358. 22615321

[2] Weaver SC, Forrester NL 2015 Chikungunya: evolutionary history and recent epidemic spread. Antiviral Res 120:32–39. doi:10.1016/j.antiviral.2015.04.016. 25979669

[3] Bruyn HB, Lennette EH 1953 Western equine encephalitis in infants; a report on three cases with sequelae. Calif Med 79:362–366. 13106712 PMC1521921

[4] Villari P, Spielman A, Komar N, McDowell M, Timperi RJ 1995 The economic burden imposed by a residual case of eastern encephalitis. Am J Trop Med Hyg 52:8–13. doi:10.4269/ajtmh.1995.52.8. 7856830

[5] Lustig S, Jackson AC, Hahn CS, Griffin DE, Strauss EG, Strauss JH 1988 Molecular basis of Sindbis virus neurovirulence in mice. J Virol 62:2329–2336. doi:10.1128/JVI.62.7.2329-2336.1988. 2836615 PMC253389

[6] Tucker PC, Griffin DE 1991 Mechanism of altered Sindbis virus neurovirulence associated with a single-amino-acid change in the E2 Glycoprotein. J Virol 65:1551–1557. doi:10.1128/JVI.65.3.1551-1557.1991. 1995953 PMC239937

[7] Tucker PC, Lee SH, Bui N, Martinie D, Griffin DE 1997 Amino acid changes in the Sindbis virus E2 glycoprotein that increase neurovirulence improve entry into neuroblastoma cells. J Virol 71:6106–6112. doi:10.1128/JVI.71.8.6106-6112.1997. 9223505 PMC191871

[8] Atkins GJ, Sheahan BJ 2016 Molecular determinants of alphavirus neuropathogenesis in mice. J Gen Virol 97:1283–1296. doi:10.1099/jgv.0.000467. 27028153

[9] Tuittila M, Hinkkanen AE 2003 Amino acid mutations in the replicase protein nsP3 of Semliki Forest virus cumulatively affect neurovirulence. J Gen Virol 84:1525–1533. doi:10.1099/vir.0.18936-0. 12771422

[10] Saul S, Ferguson M, Cordonin C, Fragkoudis R, Ool M, Tamberg N, Sherwood K, Fazakerley JK, Merits A 2015 Differences in processing determinants of nonstructural polyprotein and in the sequence of nonstructural protein 3 affect neurovirulence of Semliki Forest virus. J Virol 89:11030–11045. doi:10.1128/JVI.01186-15. 26311875 PMC4621116

[11] Gotte B, Liu L, McInerney GM 2018 The enigmatic alphavirus nonstructural protein 3 (nsP3) revealing its secrets at last. Viruses 10:e105. doi:10.3390/v10030105. 29495654 PMC5869498

[12] Voss JE, Vaney MC, Duquerroy S, Vonrhein C, Girard-Blanc C, Crublet E, Thompson A, Bricogne G, Rey FA 2010 Glycoprotein organization of chikungunya virus particles revealed by X-ray crystallography. Nature 468:709–712. doi:10.1038/nature09555. 21124458

[13] Neuvonen M, Kazlauskas A, Martikainen M, Hinkkanen A, Ahola T, Saksela K 2011 SH3 domain-mediated recruitment of host cell amphiphysins by alphavirus nsP3 promotes viral RNA replication. PLoS Pathog 7:e1002383. doi:10.1371/journal.ppat.1002383. 22114558 PMC3219718

[14] Mazzon M, Castro C, Thaa B, Liu L, Mutso M, Liu X, Mahalingam S, Griffin JL, Marsh M, McInerney GM 2018 Alphavirus-induced hyperactivation of PI3K/AKT directs pro-viral metabolic changes. PLoS Pathog 14:e1006835. doi:10.1371/journal.ppat.1006835. 29377936 PMC5805360

[15] Park E, Griffin DE 2009 Interaction of Sindbis virus nonstructural protein 3 with poly(ADP-ribose) polymerase 1 in neuronal cells. J Gen Virol 90:2073–2080. doi:10.1099/vir.0.012682-0. 19515826 PMC2887572

[16] Panas MD, Schulte T, Thaa B, Sandalova T, Kedersha N, Achour A, McInerney GM 2015 Viral and cellular proteins containing FGDF motifs bind G3BP to block stress granule formation. PLoS Pathog 11:e1004659. doi:10.1371/journal.ppat.1004659. 25658430 PMC4450067

[17] Kim DY, Reynaud JM, Rasalouskaya A, Akhrymuk I, Mobley JA, Frolov I, Frolova EI 2016 New World and Old World alphaviruses have evolved to exploit different components of stress granules, FXR and G3BP proteins, for assembly of viral replication complexes. PLoS Pathog 12:e1005810. doi:10.1371/journal.ppat.1005810. 27509095 PMC4980055

[18] Frolov I, Kim DY, Akhrymuk M, Mobley JA, Frolova EI 2017 Hypervariable domain of eastern equine encephalitis virus nsP3 redundantly utilizes multiple cellular proteins for replication complex assembly. J Virol 91:e00371-17. 28468889 10.1128/JVI.00371-17 PMC5487569

[19] LaStarza MW, Lemm JA, Rice CM 1994 Genetic analysis of the nsP3 region of Sindbis virus: evidence for roles in minus-strand and subgenomic RNA synthesis. J Virol 68:5781–5791. doi:10.1128/JVI.68.9.5781-5791.1994. 8057460 PMC236982

[20] Wang YF, Sawicki SG, Sawicki DL 1994 Alphavirus nsP3 functions to form replication complexes transcribing negative-strand RNA. J Virol 68:6466–6475. doi:10.1128/JVI.68.10.6466-6475.1994. 8083984 PMC237067

[21] Shin G, Yost SA, Miller MT, Elrod EJ, Grakoui A, Marcotrigiano J 2012 Structural and functional insights into alphavirus polyprotein processing and pathogenesis. Proc Natl Acad Sci U S A 109:16534–16539. doi:10.1073/pnas.1210418109. 23010928 PMC3478664

[22] Gao Y, Goonawardane N, Ward J, Tuplin A, Harris M 2019 Multiple roles of the nonstructural protein 3 (nsP3) alphavirus unique domain (AUD) during Chikungunya virus genome replication and transcription. PLoS Pathog 15:e1007239. doi:10.1371/journal.ppat.1007239. 30668592 PMC6358111

[23] Lykouras MV, Tsika AC, Lichiere J, Papageorgiou N, Coutard B, Bentrop D, Spyroulias GA 2018 NMR study of nonstructural proteins. III. ^1^H, ^13^C, ^15^N backbone and side-chain resonance assignment of macro domain from Chikungunya virus (CHIKV). Biomol NMR Assign 12:31–35. doi:10.1007/s12104-017-9775-2. 28875416

[24] Melekis E, Tsika AC, Lichiere J, Chasapis CT, Margiolaki I, Papageorgiou N, Coutard B, Bentrop D, Spyroulias GA 2015 NMR study of nonstructural proteins. I. ^1^H, ^13^C, ^15^N backbone and side-chain resonance assignment of macro domain from Mayaro virus (MAYV. ). Biomol NMR Assign 9:191–195. doi:10.1007/s12104-014-9572-0. 25217003

[25] Koonin EV, Gorbalenya AE, Purdy MA, Rozanov MN, Reyes GR, Bradley DW 1992 Computer-assisted assignment of functional domains in the nonstructural polyprotein of hepatitis E virus: delineation of an additional group of positive-strand RNA plant and animal viruses. Proc Natl Acad Sci U S A 89:8259–8263. doi:10.1073/pnas.89.17.8259. 1518855 PMC49897

[26] Rack JG, Perina D, Ahel I 2016 Macrodomains: structure, function, evolution, and catalytic activities. Annu Rev Biochem 85:431–454. doi:10.1146/annurev-biochem-060815-014935. 26844395

[27] Malet H, Coutard B, Jamal S, Dutartre H, Papageorgiou N, Neuvonen M, Ahola T, Forrester N, Gould EA, Lafitte D, Ferron F, Lescar J, Gorbalenya AE, de Lamballerie X, Canard B 2009 The crystal structures of Chikungunya and Venezuelan equine encephalitis virus nsP3 macro domains define a conserved adenosine binding pocket. J Virol 83:6534–6545. doi:10.1128/JVI.00189-09. 19386706 PMC2698539

[28] Egloff MP, Malet H, Putics A, Heinonen M, Dutartre H, Frangeul A, Gruez A, Campanacci V, Cambillau C, Ziebuhr J, Ahola T, Canard B 2006 Structural and functional basis for ADP-ribose and poly(ADP-ribose) binding by viral macro domains. J Virol 80:8493–8502. doi:10.1128/JVI.00713-06. 16912299 PMC1563857

[29] Neuvonen M, Ahola T 2009 Differential activities of cellular and viral macro domain proteins in binding of ADP-ribose metabolites. J Mol Biol 385:212–225. doi:10.1016/j.jmb.2008.10.045. 18983849 PMC7094737

[30] Hottiger MO, Hassa PO, Luscher B, Schuler H, Koch-Nolte F 2010 Toward a unified nomenclature for mammalian ADP-ribosyltransferases. Trends Biochem Sci 35:208–219. doi:10.1016/j.tibs.2009.12.003. 20106667

[31] Gibson BA, Kraus WL 2012 New insights into the molecular and cellular functions of poly(ADP-ribose) and PARPs. Nat Rev Mol Cell Biol 13:411–424. doi:10.1038/nrm3376. 22713970

[32] Koch-Nolte F, Kernstock S, Mueller-Dieckmann C, Weiss MS, Haag F 2008 Mammalian ADP-ribosyltransferases and ADP-ribosylhydrolases. Front Biosci 13:6716–6729. doi:10.2741/3184. 18508690

[33] Ueda K, Hayaishi O 1985 ADP-ribosylation. Annu Rev Biochem 54:73–100. doi:10.1146/annurev.bi.54.070185.000445. 3927821

[34] Verheugd P, Forst AH, Milke L, Herzog N, Feijs KL, Kremmer E, Kleine H, Luscher B 2013 Regulation of NF-κB signaling by the mono-ADP-ribosyltransferase ARTD10. Nat Commun 4:1683. doi:10.1038/ncomms2672. 23575687

[35] Gorbalenya AE, Koonin EV, Lai MM 1991 Putative papain-related thiol proteases of positive-strand RNA viruses. Identification of rubi- and aphthovirus proteases and delineation of a novel conserved domain associated with proteases of rubi-, alpha-, and coronaviruses. FEBS Lett 288:201–205. doi:10.1016/0014-5793(91)81034-6. 1652473 PMC7130274

[36] Parvez MK 2015 The hepatitis E virus ORF1 ‘X-domain’ residues form a putative macrodomain protein/Appr-1″-pase catalytic-site, critical for viral RNA replication. Gene 566:47–53. doi:10.1016/j.gene.2015.04.026. 25870943 PMC7127128

[37] Abraham R, Hauer D, McPherson RL, Utt A, Kirby IT, Cohen MS, Merits A, Leung AKL, Griffin DE 2018 ADP-ribosyl-binding and hydrolase activities of the alphavirus nsP3 macrodomain are critical for initiation of virus replication. Proc Natl Acad Sci U S A 115:E10457–E10466. doi:10.1073/pnas.1812130115. 30322911 PMC6217424

[38] Eriksson KK, Cervantes-Barragan L, Ludewig B, Thiel V 2008 Mouse hepatitis virus liver pathology is dependent on ADP-ribose-1″-phosphatase, a viral function conserved in the alpha-like supergroup. J Virol 82:12325–12334. doi:10.1128/JVI.02082-08. 18922871 PMC2593347

[39] Fehr AR, Athmer J, Channappanavar R, Phillips JM, Meyerholz DK, Perlman S 2015 The nsp3 macrodomain promotes virulence in mice with coronavirus-induced encephalitis. J Virol 89:1523–1536. doi:10.1128/JVI.02596-14. 25428866 PMC4300739

[40] Fehr AR, Channappanavar R, Jankevicius G, Fett C, Zhao J, Athmer J, Meyerholz DK, Ahel I, Perlman S 2016 The conserved coronavirus macrodomain promotes virulence and suppresses the innate immune response during severe acute respiratory syndrome coronavirus infection. mBio 7:e01721-16. doi:10.1128/mBio.01721-16. 27965448 PMC5156301

[41] Jankevicius G, Hassler M, Golia B, Rybin V, Zacharias M, Timinszky G, Ladurner AG 2013 A family of macrodomain proteins reverses cellular mono-ADP-ribosylation. Nat Struct Mol Biol 20:508–514. doi:10.1038/nsmb.2523. 23474712 PMC7097781

[42] Rosenthal F, Feijs KL, Frugier E, Bonalli M, Forst AH, Imhof R, Winkler HC, Fischer D, Caflisch A, Hassa PO, Luscher B, Hottiger MO 2013 Macrodomain-containing proteins are new mono-ADP-ribosylhydrolases. Nat Struct Mol Biol 20:502–507. doi:10.1038/nsmb.2521. 23474714

[43] McPherson RL, Abraham R, Sreekumar E, Ong SE, Cheng SJ, Baxter VK, Kistemaker HA, Filippov DV, Griffin DE, Leung AK 2017 ADP-ribosylhydrolase activity of Chikungunya virus macrodomain is critical for virus replication and virulence. Proc Natl Acad Sci U S A 114:1666–1671. doi:10.1073/pnas.1621485114. 28143925 PMC5321000

[44] Li C, Debing Y, Jankevicius G, Neyts J, Ahel I, Coutard B, Canard B 2016 Viral macro domains reverse protein ADP-ribosylation. J Virol 90:8478–8486. doi:10.1128/JVI.00705-16. 27440879 PMC5021415

[45] Eckei L, Krieg S, Butepage M, Lehmann A, Gross A, Lippok B, Grimm AR, Kummerer BM, Rossetti G, Luscher B, Verheugd P 2017 The conserved macrodomains of the nonstructural proteins of Chikungunya virus and other pathogenic positive strand RNA viruses function as mono-ADP-ribosylhydrolases. Sci Rep 7:41746. doi:10.1038/srep41746. 28150709 PMC5288732

[46] Atasheva S, Akhrymuk M, Frolova EI, Frolov I 2012 New PARP gene with an anti-alphavirus function. J Virol 86:8147–8160. doi:10.1128/JVI.00733-12. 22623789 PMC3421642

[47] Daugherty MD, Young JM, Kerns JA, Malik HS 2014 Rapid evolution of PARP genes suggests a broad role for ADP-ribosylation in host-virus conflicts. PLoS Genet 10:e1004403. doi:10.1371/journal.pgen.1004403. 24875882 PMC4038475

[48] Kerns JA, Emerman M, Malik HS 2008 Positive selection and increased antiviral activity associated with the PARP-containing isoform of human zinc-finger antiviral protein. PLoS Genet 4:e21. doi:10.1371/journal.pgen.0040021. 18225958 PMC2213710

[49] Goodier JL, Pereira GC, Cheung LE, Rose RJ, Kazazian HH Jr. 2015 The broad-spectrum antiviral protein ZAP restricts human retrotransposition. PLoS Genet 11:e1005252. doi:10.1371/journal.pgen.1005252. 26001115 PMC4441479

[50] Leung AKL, McPherson RL, Griffin DE 2018 Macrodomain ADP-ribosylhydrolase and the pathogenesis of infectious diseases. PLoS Pathog 14:e1006864. doi:10.1371/journal.ppat.1006864. 29566066 PMC5864081

[51] Fehr AR, Jankevicius G, Ahel I, Perlman S 2018 Viral macrodomains: unique mediators of viral replication and pathogenesis. Trends Microbiol 26:598–610. doi:10.1016/j.tim.2017.11.011. 29268982 PMC6003825

[52] Tucker PC, Strauss EG, Kuhn RJ, Strauss JH, Griffin DE 1993 Viral determinants of age-dependent virulence of Sindbis virus for mice. J Virol 67:4605–4610. doi:10.1128/JVI.67.8.4605-4610.1993. 8392602 PMC237845

[53] Baxter VK, Glowinski R, Braxton AM, Potter MC, Slusher BS, Griffin DE 2017 Glutamine antagonist-mediated immune suppression decreases pathology but delays virus clearance in mice during nonfatal alphavirus encephalomyelitis. Virology 508:134–149. doi:10.1016/j.virol.2017.05.013. 28531865 PMC5510753

[54] Griffin DE, Levine B, Tyor WR, Irani DN 1992 The immune response in viral encephalitis. Semin Immunol 4:111–119. 1319767

[55] Park E, Griffin DE 2009 The nsP3 macro domain is important for Sindbis virus replication in neurons and neurovirulence in mice. Virology 388:305–314. doi:10.1016/j.virol.2009.03.031. 19395054 PMC2683903

[56] Akhrymuk I, Frolov I, Frolova EI 2018 Sindbis virus infection causes cell death by nsP2-induced transcriptional shutoff or by nsP3-dependent translational shutoff. J Virol 92:e01388-18. doi:10.1128/JVI.01388-18. 30232189 PMC6232463

[57] Rungrotmongkol T, Nunthaboot N, Malaisree M, Kaiyawet N, Yotmanee P, Meeprasert A, Hannongbua S 2010 Molecular insight into the specific binding of ADP-ribose to the nsP3 macro domains of chikungunya and Venezuelan equine encephalitis viruses: molecular dynamics simulations and free energy calculations. J Mol Graph Model 29:347–353. doi:10.1016/j.jmgm.2010.09.010. 21036084

[58] Kistemaker HA, Nardozza AP, Overkleeft HS, van der Marel GA, Ladurner AG, Filippov DV 2016 Synthesis and macrodomain binding of mono-ADP-ribosylated peptides. Angew Chem Int Ed Engl 55:10634–10638. doi:10.1002/anie.201604058. 27464500

[59] Karras GI, Kustatscher G, Buhecha HR, Allen MD, Pugieux C, Sait F, Bycroft M, Ladurner AG 2005 The macro domain is an ADP-ribose binding module. EMBO J 24:1911–1920. doi:10.1038/sj.emboj.7600664. 15902274 PMC1142602

[60] Kustatscher G, Hothorn M, Pugieux C, Scheffzek K, Ladurner AG 2005 Splicing regulates NAD metabolite binding to histone macroH2A. Nat Struct Mol Biol 12:624–625. doi:10.1038/nsmb956. 15965484

[61] Timinszky G, Till S, Hassa PO, Hothorn M, Kustatscher G, Nijmeijer B, Colombelli J, Altmeyer M, Stelzer EH, Scheffzek K, Hottiger MO, Ladurner AG 2009 A macrodomain-containing histone rearranges chromatin upon sensing PARP1 activation. Nat Struct Mol Biol 16:923–929. doi:10.1038/nsmb.1664. 19680243

[62] Wienken CJ, Baaske P, Rothbauer U, Braun D, Duhr S 2010 Protein-binding assays in biological liquids using microscale thermophoresis. Nat Commun 1:100. doi:10.1038/ncomms1093. 20981028

[63] Tan ES, Krukenberg KA, Mitchison TJ 2012 Large-scale preparation and characterization of poly(ADP-ribose) and defined length polymers. Anal Biochem 428:126–136. doi:10.1016/j.ab.2012.06.015. 22743307 PMC3414684

[64] Gao G, Guo X, Goff SP 2002 Inhibition of retroviral RNA production by ZAP, a CCCH-type zinc finger protein. Science 297:1703–1706. doi:10.1126/science.1074276. 12215647

[65] Grunewald ME, Chen Y, Kuny C, Maejima T, Lease R, Ferraris D, Aikawa M, Sullivan CS, Perlman S, Fehr AR 2019 The coronavirus macrodomain is required to prevent PARP-mediated inhibition of virus replication and enhancement of IFN expression. PLoS Pathog 15:e1007756. doi:10.1371/journal.ppat.1007756. 31095648 PMC6521996

[66] Shaw AE, Hughes J, Gu Q, Behdenna A, Singer JB, Dennis T, Orton RJ, Varela M, Gifford RJ, Wilson SJ, Palmarini M 2017 Fundamental properties of the mammalian innate immune system revealed by multispecies comparison of type I interferon responses. PLoS Biol 15:e2004086. doi:10.1371/journal.pbio.2004086. 29253856 PMC5747502

[67] Zhang Y, Burke CW, Ryman KD, Klimstra WB 2007 Identification and characterization of interferon-induced proteins that inhibit alphavirus replication. J Virol 81:11246–11255. doi:10.1128/JVI.01282-07. 17686841 PMC2045553

[68] Atasheva S, Frolova EI, Frolov I 2014 Interferon-stimulated poly(ADP-Ribose) polymerases are potent inhibitors of cellular translation and virus replication. J Virol 88:2116–2130. doi:10.1128/JVI.03443-13. 24335297 PMC3911523

[69] Welsby I, Hutin D, Gueydan C, Kruys V, Rongvaux A, Leo O 2014 PARP12, an interferon-stimulated gene involved in the control of protein translation and inflammation. J Biol Chem 289:26642–26657. doi:10.1074/jbc.M114.589515. 25086041 PMC4176246

[70] Wang N, Dong Q, Li J, Jangra RK, Fan M, Brasier AR, Lemon SM, Pfeffer LM, Li K 2010 Viral induction of the zinc finger antiviral protein is IRF3-dependent but NF-κB-independent. J Biol Chem 285:6080–6090. doi:10.1074/jbc.M109.054486. 20048147 PMC2825402

[71] Garcia-Sastre A, Biron CA 2006 Type 1 interferons and the virus-host relationship: a lesson in detente. Science 312:879–882. doi:10.1126/science.1125676. 16690858

[72] Shuai K, Schindler C, Prezioso VR, Darnell JE Jr. 1992 Activation of transcription by IFN-gamma: tyrosine phosphorylation of a 91-kD DNA binding protein. Science 258:1808–1812. doi:10.1126/science.1281555. 1281555

[73] Baxter VK, Troisi EM, Pate NM, Zhao JN, Griffin DE 2018 Death and gastrointestinal bleeding complicate encephalomyelitis in mice with delayed appearance of CNS IgM after intranasal alphavirus infection. J Gen Virol doi:10.1099/jgv.0.001005. 10.1099/jgv.0.001005 PMC5891226 29458665

[74] Burdeinick-Kerr R, Wind J, Griffin DE 2007 Synergistic roles of antibody and interferon in noncytolytic clearance of Sindbis virus from different regions of the central nervous system. J Virol 81:5628–5636. doi:10.1128/JVI.01152-06. 17376910 PMC1900320

[75] Binder GK, Griffin DE 2001 Interferon-gamma-mediated site-specific clearance of alphavirus from CNS neurons. Science 293:303–306. doi:10.1126/science.1059742. 11452126

[76] Kulcsar KA, Griffin DE 2016 T cell-derived interleukin-10 is an important regulator of the Th17 response during lethal alphavirus encephalomyelitis. J Neuroimmunol 295–296:60–67. doi:10.1016/j.jneuroim.2016.04.010. PMC4884611 27235350

[77] Byrnes AP, Durbin JE, Griffin DE 2000 Control of Sindbis virus infection by antibody in interferon-deficient mice. J Virol 74:3905–3908. doi:10.1128/jvi.74.8.3905-3908.2000. 10729167 PMC111901

[78] Ryman KD, Klimstra WB, Nguyen KB, Biron CA, Johnston RE 2000 Alpha/beta interferon protects adult mice from fatal Sindbis virus infection and is an important determinant of cell and tissue tropism. J Virol 74:3366–3378. doi:10.1128/jvi.74.7.3366-3378.2000. 10708454 PMC111838

[79] Ryman KD, Meier KC, Gardner CL, Adegboyega PA, Klimstra WB 2007 Non-pathogenic Sindbis virus causes hemorrhagic fever in the absence of alpha/beta and gamma interferons. Virology 368:273–285. doi:10.1016/j.virol.2007.06.039. 17681583

[80] Kuri T, Eriksson KK, Putics A, Zust R, Snijder EJ, Davidson AD, Siddell SG, Thiel V, Ziebuhr J, Weber F 2011 The ADP-ribose-1″-monophosphatase domains of severe acute respiratory syndrome coronavirus and human coronavirus 229E mediate resistance to antiviral interferon responses. J Gen Virol 92:1899–1905. doi:10.1099/vir.0.031856-0. 21525212

[81] Nan Y, Yu Y, Ma Z, Khattar SK, Fredericksen B, Zhang YJ 2014 Hepatitis E virus inhibits type I interferon induction by ORF1 products. J Virol 88:11924–11932. doi:10.1128/JVI.01935-14. 25100852 PMC4178743

[82] Levine B, Hardwick JM, Trapp BD, Crawford TO, Bollinger RC, Griffin DE 1991 Antibody-mediated clearance of alphavirus infection from neurons. Science 254:856–860. doi:10.1126/science.1658936. 1658936

[83] Burdeinick-Kerr R, Griffin DE 2005 Gamma interferon-dependent, noncytolytic clearance of Sindbis virus infection from neurons *in vitro*. J Virol 79:5374–5385. doi:10.1128/JVI.79.9.5374-5385.2005. 15827152 PMC1082728

[84] Hardwick JM, Levine B 2000 Sindbis virus vector system for functional analysis of apoptosis regulators. Methods Enzymol 322:492–508. doi:10.1016/s0076-6879(00)22045-4. 10914042

[85] Chollet A, Kawashima EH 1985 Biotin-labeled synthetic oligodeoxyribonucleotides: chemical synthesis and uses as hybridization probes. Nucleic Acids Res 13:1529–1541. doi:10.1093/nar/13.5.1529. 4000941 PMC341094

[86] Ando Y, Elkayam E, McPherson RL, Dasovich M, Cheng SJ, Voorneveld J, Filippov DV, Ong SE, Joshua-Tor L, Leung A 2019 ELTA: enzymatic labeling of terminal ADP-ribose. Mol Cell 73:846–856. 10.1016/j.molcel.2018.12.022 PMC6629254 30712989

[87] Cashman NR, Durham HD, Blusztajn JK, Oda K, Tabira T, Shaw IT, Dahrouge S, Antel JP 1992 Neuroblastoma × spinal cord (NSC) hybrid cell lines resemble developing motor neurons. Dev Dyn 194:209–221. doi:10.1002/aja.1001940306. 1467557

[88] Durham HD, Dahrouge S, Cashman NR 1993 Evaluation of the spinal cord neuron X neuroblastoma hybrid cell line NSC-34 as a model for neurotoxicity testing. Neurotoxicology 14:387–395. 7909362

[89] Nilaratanakul V, Chen J, Tran O, Baxter VK, Troisi EM, Yeh JX, Griffin DE 2018 Germ line IgM is sufficient, but not required, for antibody-mediated alphavirus clearance from the central nervous system. J Virol 92:e02081-17. doi:10.1128/JVI.02081-17. 29321331 PMC5972861

[90] Goenka S, Boothby M 2006 Selective potentiation of Stat-dependent gene expression by collaborator of Stat6 (CoaSt6), a transcriptional cofactor. Proc Natl Acad Sci U S A 103:4210–4215. doi:10.1073/pnas.0506981103. 16537510 PMC1449672

